# Antagonism of propofol anesthesia by alkyl-fluorobenzene derivatives

**DOI:** 10.1038/s41598-024-66672-z

**Published:** 2024-07-10

**Authors:** Diana M. Plasencia, Liam H. Rodgers, Alexys R. Knighton, Roderic G. Eckenhoff, E. Railey White

**Affiliations:** Department of Anesthesiology and Critical Care, Perelman School of Medicine, University of Pennsylvania, Philadelphia, USA

**Keywords:** Structure-based drug design, Pharmacology, Small molecules, Consciousness

## Abstract

Despite their frequent use across many clinical settings, general anesthetics are medications with lethal side effects and no reversal agents. A fluorinated analogue of propofol has previously been shown to antagonize propofol anesthesia in tadpoles and zebrafish, but little further investigation of this class of molecules as anesthetic antagonists has been conducted. A 13-member library of alkyl-fluorobenzene derivatives was tested in an established behavioral model of anesthesia in zebrafish at 5 days post fertilization. These compounds were examined for their ability to antagonize propofol and two volatile anesthetics, as well as their interaction with the anesthetic-binding model protein apoferritin. Two compounds provided significant antagonism of propofol, and when combined, were synergistic, suggesting more than one antagonist sensitive target site. These compounds did not antagonize the volatile anesthetics, indicating some selectivity amongst general anesthetics. For the compounds with the most antagonistic potency, similarities in structure and binding to apoferritin may be suggestive of competitive antagonism; however, this was not supported by a Schild analysis. This is consistent with multiple targets contributing to general anesthesia, but whether these are physiologic antagonists or are antagonists at only some subset of the many anesthetic potential targets remains unclear, and will require additional investigation.

## Introduction

Propofol is widely used for induction of anesthesia and has gained increasing popularity as a maintenance anesthetic for many procedures, from colonoscopies to craniotomies, as well as sedation in intensive care units (ICUs)^1^. The benefits of propofol include its relatively fast onset and offset of action as well as a comparatively low side-effect profile; however, propofol has non-anesthetic effects (including cardiovascular and respiratory depression) that can result in catastrophe if not anticipated and mitigated. One approach to mitigation would be drug antagonists. With the introduction of sugammadex, there are now antagonists for every class of commonly used perioperative drug except the general anesthetics^2^. There are many clinical settings in which an anesthetic antagonist could be useful. In addition to saving time during emergence, an antagonist could be lifesaving when seconds count, such as the dreaded ‘can’t ventilate, can’t intubate’ scenario. Further, an antagonist may facilitate critical neurological evaluations in the ICU, and obviate or expedite the need for a CT scan to differentiate a stroke from residual anesthesia. These are just a few examples where the availability of an anesthetic antagonist could change clinical practice, and thus is not surprising that the development of anesthetic antagonists is an area of increasing interest^3^.

Prior efforts to identify possible anesthetic antagonists have suffered from an incomplete knowledge of anesthetic molecular pharmacology^4^. Given that many anesthetics activate the GABA_A_ receptor (GABA_A_R), rational drug design of GABA_A_R inhibitors have been pursued with some success^5^, but translation to in vivo anesthetic antagonism is lacking. Others have focused on non-specific CNS stimulants (caffeine, methylphenidate, etc.) to hasten the emergence from anesthetics^6–8^, though undesirable side effects (dysrhythmias, hemodynamic extremes, delirium, psychosis, etc.) can occur^9,10^. Furthermore, these nonspecific drugs are less effective for propofol, the most commonly used anesthetic for induction and sedation. An effective antagonist specific to propofol with few side effects would be clinically useful, and a logical approach may be to explore propofol derivatives as potential competitive antagonists.

The study of anesthetic structure activity relationships (SARs) dates back to Meyer and Overton in the 1880s and has continued as a means of anesthetic optimization, as well as to probe potential anesthetic mechanisms^11,12^. Propofol derivatives have previously been classified as either “active” or “inactive” as anesthetics, where the inactive ones are disregarded^13,14^. But a lack of anesthetic activity does not necessarily correspond to lack of any pharmacologic activity. For example, the molecule Fropofol (fluorinated propofol)^15^, (renamed propofluor for improved phonological clarity), is one such molecule. Although propofluor is inactive as an anesthetic, it was found to antagonize propofol in tadpoles^15^ and zebrafish^16^. This study examines alkyl variations of propofluor for anesthetic and antagonistic properties using a previously validated zebrafish behavioral model of anesthesia^17,18^.

## Results

### Selection of library compounds

**Figure 1:**
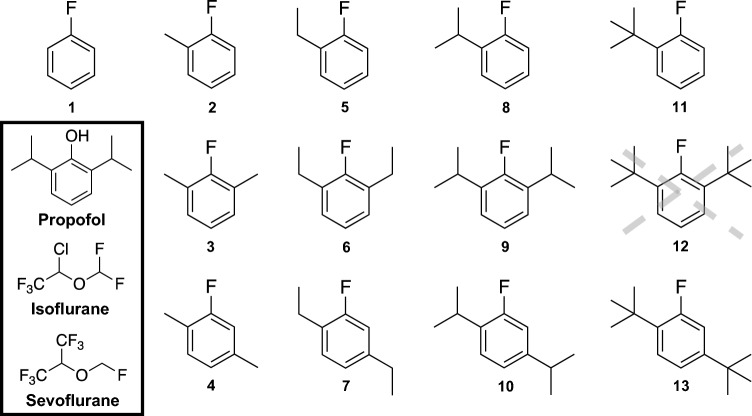
Chemical Structures of the Propofluor Arm Library (PFAL) Compounds and Commonly Used Anesthetics. This library of compounds is composed of alkyl derivatives of the molecule propofluor (compound **9**, previously called fropofol). The intended compound **12** was unable to be synthesized and thus not characterized. Chemical names and abbreviations are as follows: **1**: fluorobenzene (FB), **2**: 1-methyl-2-fluorobenzene (1-Me-2-FB), **3**: 1,3-dimethyl-2-fluorobenzene (1,3-diMe-2-FB), **4**: 1,4-dimethyl-2-fluorobenzene (1,4-diMe-2-FB), **5**: 1-ethyl-2-fluorobenzene (1-Et-2-FB), **6**: 1,3-diethyl-2-fluorobenzene (1,3-diEt-2-FB), **7**: 1,4-diethyl-2-fluorobenzene (1,4-diEt-2-FB), **8**: 1-isopropyl-2-fluorobenzene (1-iPr-2-FB), **9**: 1,3-diisopropyl-2-fluorobenzene (1,3-diiPr-FB) **10**: 1,4-diisopropyl-2-fluorobenzene (1,4-diiPr-FB), **11**: 1-tertbutyl-2-fluorobenzene (1-ditBu-FB), **12**: 1,3-ditertbutyl-2-fluorobenzene (1,3-ditBu-FB), **13**: 1,4-ditertbutyl-2-fluorobenzene (1,4-ditBu-FB). The chemical structures of 3 commonly used anesthetics (Propofol, Sevoflurane and Isoflurane) are shown in the lower left corner.

All compounds for the propofluor arm library (PFAL) were designed around a fluorobenzene core, with variable alkyl ‘arms’. One could generate a massive library solely with modifications to alkyl carbon number, position, and shape, but the final 13-member PFAL (Fig. 1) consisted of a subset of non-chiral alkyl arms (methyl, ethyl, isopropyl and tertbutyl) in three different positions (1, 1,3 and 1,4) on the fluorobenzene ring to create a library with systematic alterations in size and configuration of the alkyl carbon side chains. The proposed compound **12** (1,3-ditertbutyl-2-fluorobenzene) was unable to be synthesized and thus not included in the library. Physiochemical parameters for these compounds can be found in Table S1.

### Toxicity of PFAL compounds

An important initial step in the testing these compounds is an assessment of the toxicity. The small size of 5 dpf (days post fertilization) larval zebrafish (< 4 mm total body length) facilitates bulk diffusion for gas transport, thus toxicity due to respiratory depression is inaccessible and therefore a limitation of this model compared to adult fish and mammals that rely on breathing and bulk flow respiration^19,20^. Nevertheless, an LD_50_ (median lethal dose) in this model remains a useful means to compare toxicity, and also prevents mistakenly characterizing mortality as the decreased movement observed with anesthetic states. In this assay, PFAL compounds were found to be considerably less toxic than propofol itself (Fig. S1), including some with LD_50_ values that were unable to be determined due to both low toxicity and limited solubility. For these compounds (**1**–**5**) an LD_50_ was estimated with available experimental data and an upper constraint of 100% mortality. All concentrations of PFAL compounds used in later testing were far below these LD_50_ values. Co-administration 30 μM PFAL compounds with propofol yielded a minimal increase (< 6 μM changes) in LD_50_ that remains far above propofol’s clinically useful concentrations.

### Activity of the PFAL compounds

**Figure 2:**
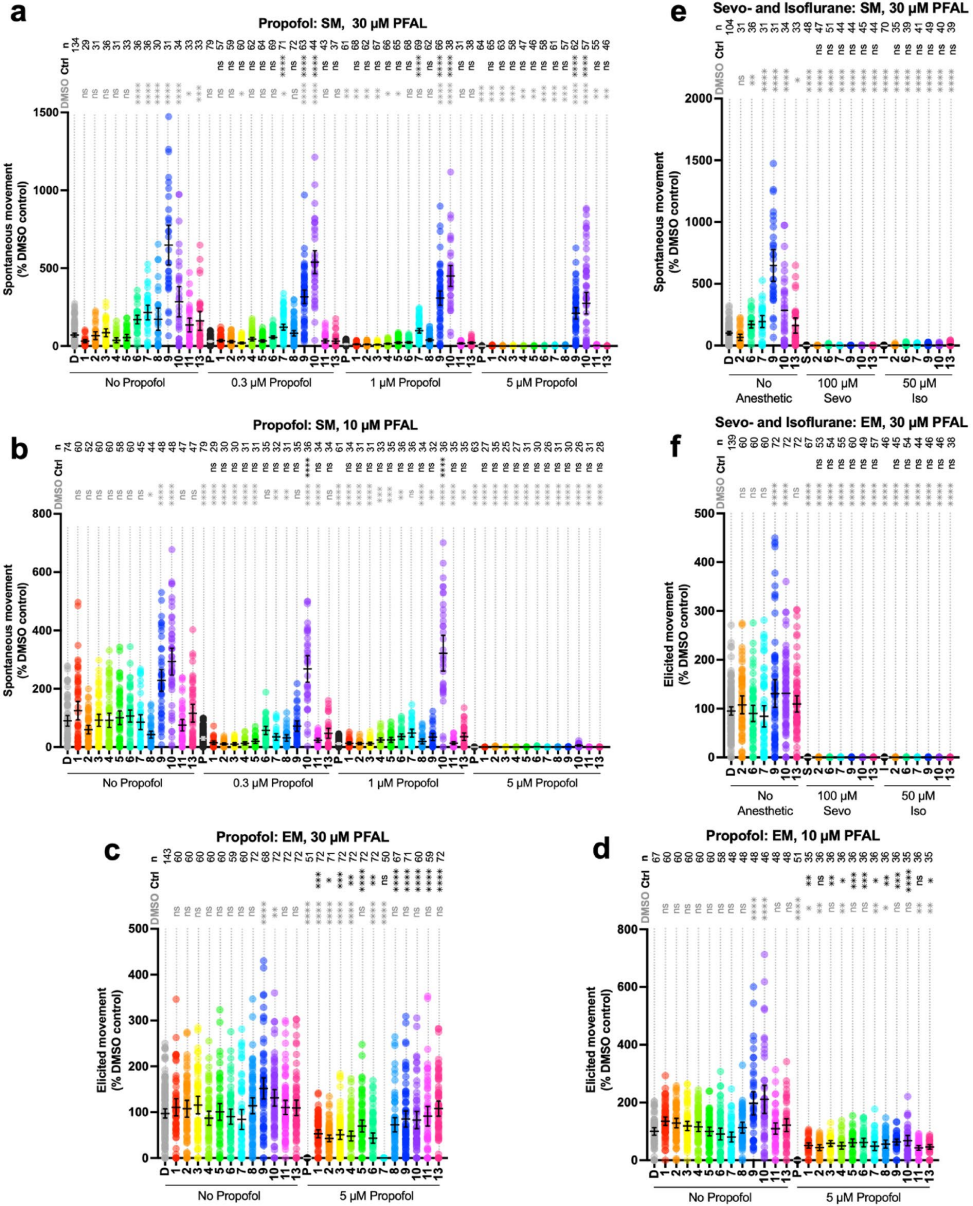
Screening of PFAL compounds for antagonism of anesthesia. PFAL compounds were screened in 5 dpf zebrafish for their ability to antagonize the anesthetic effects of propofol (panels a, b, c and d) as well as sevoflurane and isoflurane (panels e and f). Each dot represents the data collected from one fish. Spontaneous movement (SM) is the distance of spontaneous swimming in a 4 min period, and elicited movement (EM) is distance moved in 1 s after exposure to a tap stimulus. Both measures were scaled to same-day DMSO controls. Concentrations of drugs used for screening were based on previously determined EC_50_ values: Propofol SM = 0.01 μM, EM= 2.8 μM; Sevoflurane SM = 76 μM, EM = 240 μM; Isoflurane SM = 42, μM EM = 180 μM^17^. Mean and 95% CI are shown for each data set. Statistical comparison with both DMSO (D) only (black asterisks) and anesthetic control (Ctrl, gray asterisks) groups are shown. Note that each DMSO comparison is to the DMSO only control (sample on the furthest left of each graph, and the anesthetic control comparisons are made within each anesthetic grouping as indicated by the bars along the x-axis of each graph. ns: *P* > 0.05, *: *P* ≤ 0.05, **: *P* ≤ 0.01, ***: *P* ≤ 0.001, ****: *P* ≤ 0.0001).

Each compound was tested for sedative activity when administered alone prior to combination testing with propofol (Fig. 2a-d). PFAL compounds alone at 10 and 30 μM revealed no statistically significant decrease in spontaneous movement (SM) or elicited movement (EM) that would indicate sedation or anesthesia. Although no decrease in SM or EM was observed for any PFAL compound, this does not rule out sub-clinical sedation that might be observed with propofol co-administration. On the other hand, compounds **7**, **9**, and **10** demonstrated increased SM, and only compounds **9** and **10** showed increased EM. To further characterize this increase in SM/EM, we compared the pattern of activity to that produced by pentylenetetrazole^21^, a known epileptogenic compound, and the observed increase in SM was not consistent with seizure. Pentylenetetrazole administration results in a period of normal movement, followed by a short period of uncontrolled movement (seizure) and then decreased movement characteristic of a post-ictal state. This is in contrast to the PFAL treated fish where this dose-dependent increase in movement was simply more rapid movement (Fig. S2 and Supplemental Video). A change in movement due to chemical irritation is another consideration, but previous data using mustard oil shows that chemical irritation actually results in decreased movement^17^. Ultimately, the mechanism of the observed increase in movement is not clear but may relate to activation of specific motor networks.

### Antagonism of propofol anesthesia

Based on previously reported EC_50_ values of propofol in 5 dpf zebrafish (propofol EC_50_ for SM = 0.1 μM, EC_50_ for EM = 3.8 μM)^17^, propofol concentrations of 0.3, 1, and 5 μM were co-administered with PFAL compounds (up to 30 μM) as an initial test of anesthetic additivity or antagonism. Compounds **7**, **9** and **10** administered at 30 μM showed antagonism of propofol via measures of SM, but only compounds **9** and **10** antagonized propofol at a concentration of 5 μM (Fig. 2a). To further test the potency of these compounds, the PFAL concentration was tested at 10 μM and with this lower dose only **10** demonstrated antagonism of propofol (Fig. 2b). Despite having the most antagonistic potency for SM, even **10** did not show antagonism at a propofol concentration of 5 μM. However, at a concentration of 10 μM, **10** was able to antagonize propofol at 1 μM which is about 10-times higher than propofol’s SM EC_50_ of 0.1 μM. This initial screen seems to suggest compound **10** is more potent as a propofol antagonist compared to the previously characterized compound **9** (propofluor).

**Figure 3:**
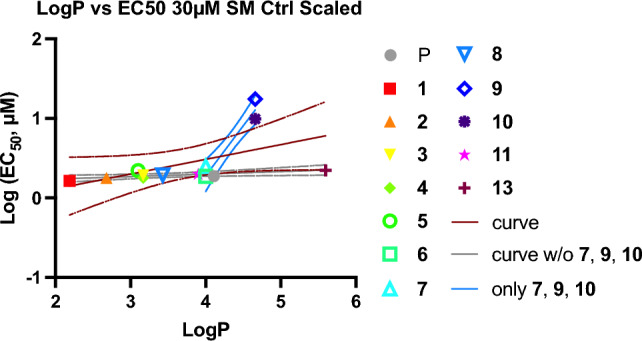
Comparison of LogP and Estimated Antagonistic Potency. The EC_50_ of propofol in the presence of PFAL compounds (as indicated in the legend) were estimated from the screening data in Fig. 2. These values were compared to calculated LogP values for each compound. Linear regressions (95% CI shown) exclude the data from propofol only (*P*). The gray regression excludes the compounds that demonstrated statistically significant antagonism via SM (**7**, **9**, and **10**) and the blue regression excludes the other compounds that did not demonstrate antagonism.

**Figure 4:**
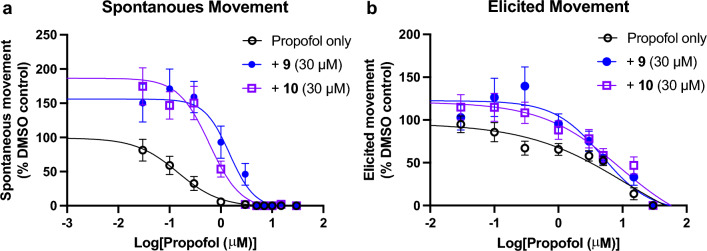
Propofol EC_50_ curves with PFAL 9 and 10. **a** Spontaneous movement of propofol alone and with the addition of 30 μM **9** or **10**. Propofol alone had an EC_50_ of 0.14 μM (95% CI 0.10–0.20), and both **9** and **10** showed statistically significant rightward shifts in EC_50_. **9**: 1.4 μM (95% CI 1.10–1.88), *P* < 0.0001 (~ tenfold increase); **10**: 0.58 μM (95% CI 0.35–0.75), *P* < 0.0001 (~ fourfold increase). **b** Elicited movement of propofol alone and with the addition of 30 μM **9** or **10**. Propofol alone had an EC_50_ of 3.53 μM (95% CI 2.29–5.13), neither **9** nor **10** showed statistically significant shifts in EC_50_. **9**: 4.38 μM (95% CI 3.22–6.06), *P* = 0.50; **10**: 5.00 μM (95% CI 2.85–5.91), *P* = 0.23.

The pattern of SM antagonism demonstrated by the PFAL compounds suggests that there may be a ‘sweet spot’ of hydrophobicity and/or sterics that makes an important contribution to antagonistic potency. It is well described that relative LogP of anesthetics correlates to potency, if hydrophobicity alone were a primary drive of potency, one would expect to see a bell-shaped trend with a peak indicating ideal configuration and/or hydrophobicity. However, an analysis of the estimated EC_50_ derived from this initial screening (see Fig. S3) compared to the calculated LogP shows little variation amongst the antagonism of PFAL compounds, with the obvious exceptions of **9** and **10** (see Fig. 3). This pattern suggests that the geometry of the carbon chains has a greater contribution to antagonist potency than simple hydrophobicity. Interestingly, although much less potent than **10** by measures of SM, **7** was the third most potent antagonizing compound, and it shares the same ‘1,4’ alkyl chain configuration as **10**. This may additionally suggest differences compared to the ‘1,3’ alkyl configuration. However, it is also notable that when examined for antagonism via measures of EM (Fig. 2c-d), **7** was the only compound that did not antagonize propofol at 30 μM PFAL concentration (Fig. 2c). Overall, there is poor correlation in this initial screen between antagonism of propofol by SM and by EM. At 30 μM concentrations for EM, all the PFAL compounds (except **7**) showed statistically significant antagonism of propofol. Even at 10 μM PFAL, only the EM of **2** and **11** were found not to be statistically different than the propofol controls. The inability to distinguish large differences in potency for EM based on structural differences supports mechanistic differences in antagonism, and by proxy differences in the underlying anesthetic mechanism of these movements. However, even with compounds that showed antagonism, this initial screen by a measure of EM (response to tap stimulus) was not found to correspond to a statistically significant rightward shift in the EC_50_ of propofol for compounds **9** and **10** (see Fig. 4). Thus, there are obvious limitations in what conclusions with respect to anesthetic antagonism can be draw from the single concentration tested in the screen, but it does not negate the interest of these findings as there are multiple important anesthetic endpoints and the antagonism of propofol via measure of SM is worth further consideration.

### Antagonism of sevoflurane and isoflurane anesthesia

To assess the selectivity of this anesthetic antagonism, a smaller group of PFAL compounds was tested for antagonism of two volatile anesthetics sevoflurane and isoflurane. As with propofol, these anesthetic concentrations were initially chosen for each drug based on previously reported EC_50_ values (Sevoflurane SM = 76 μM, EM = 240 μM; Isoflurane SM = 42 μM, EM = 180 μM)^17^, and PFAL doses of 30 μM. No evidence of antagonism of either volatile anesthetic was found (see Fig. 2e-f) via measures of SM or EM. These findings suggest some selectivity for antagonism of propofol anesthesia, and render unlikely the idea that antagonism is arising from non-specific stimulation. This is similar to previous findings that indicate the opposite; some stimulants having greater potency in antagonizing volatile anesthetics compared to propofol^8^, lending further support for underlying differences in mechanisms of action for these anesthetic classes.

### Antagonism of anesthesia after induction

**Figure 5:**
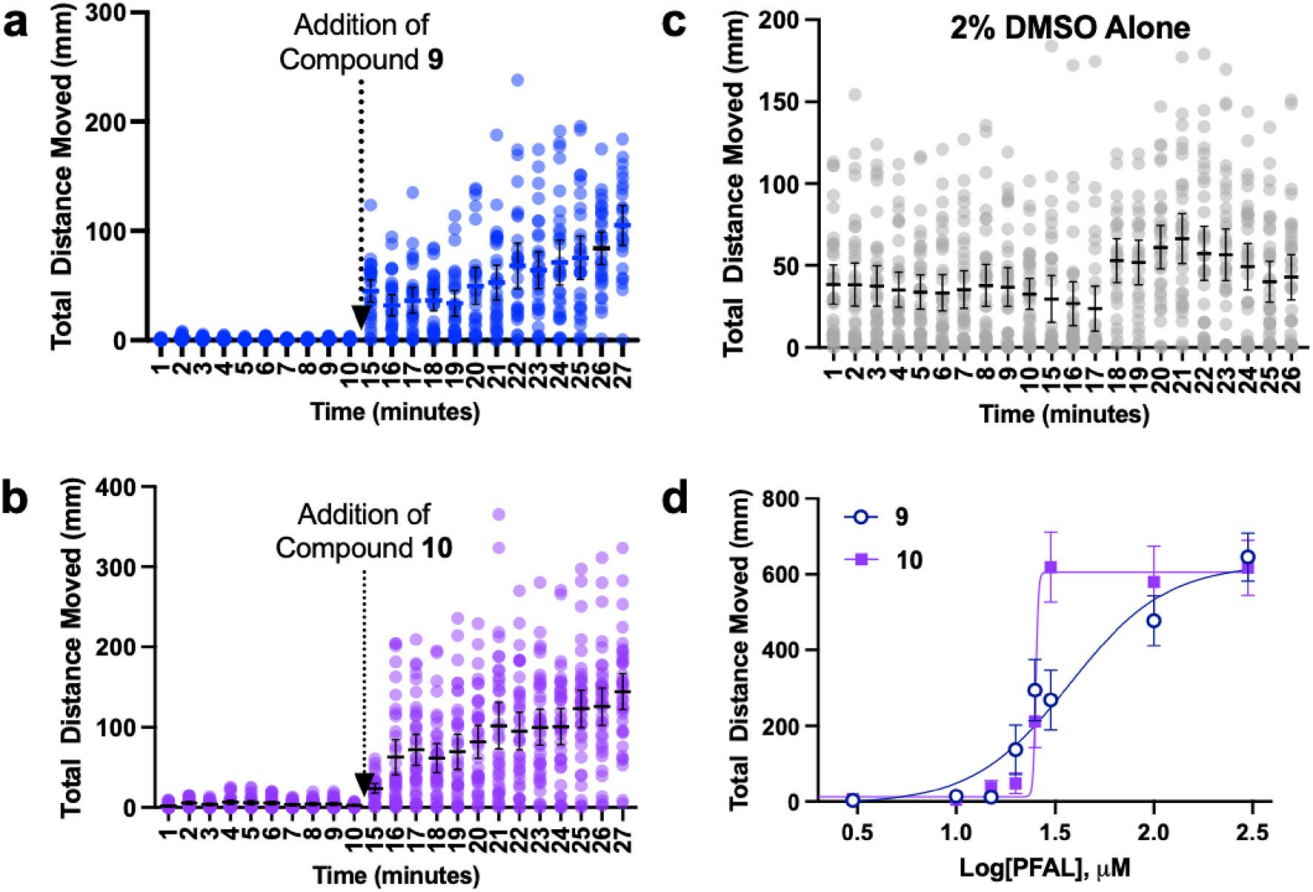
Further Characterization of Propofol Antagonism with Compounds 9 and 10. **a** and **b** 5 dpf zebrafish (n = 36) were first anesthetized with 3 μM propofol and then 30 μM of either compound **9** or **10** was added. Between minutes 5 and 10 there is a small gap in the data during which the propofol solution was removed and a solution containing propofol and PFAL compound was added. Spontaneous movement of the fish resumed very shortly after compound addition. **c** 2% DMSO vehicle control for panels A and B. n = 36 for panels A-C (each dot represents 1 fish). **d** IC_50_ of compounds **9** and **10** measured in 5 dpf zebrafish exposed to 3 μM Propofol was found to be 37.5 μM and 25.3 μM respectively. The mean and 95% CI is shown for each data point in all panels.

In the above studies, the anesthetic and PFAL compounds were co-administered to the fish simultaneously. In order to show that these compounds can antagonize ‘anesthesia’ once established, a sequential addition was conducted whereby the fish were exposed to propofol (3 μM) for 10 min, the solution was removed and replaced with solution containing propofol (3 μM) and either compound **9** or **10** (30 μM). Although this experiment does not provide precise kinetic data, the exposure to either **9** or **10** produced rapid reversal of propofol immobility despite the continued presence of an immobilizing concentration of propofol (Fig. 5a-c).

**IC**_**50**_** of compounds 9 and 10**. The initial screening experiments revealed **9** and **10** as the most potent anesthetic antagonists in the PFAL library. At a propofol concentration of 3 μM, IC_50_ (median inhibitory concentration) values were determined to quantitatively compare their potency (Fig. 5d). The IC_50_ values were closer than the initial screening experiments suggested (IC_50_ of **9** = 37.5 μM, **10** = 25.3 μM); however, the finding is less surprising when one considers the striking difference in Hill slopes (**9** = 1.8, **10** = 65), may also be an additional clue to that these compounds have mechanistic differences.

### Inhibition of 1-AMA binding to a model protein

**Figure 6:**
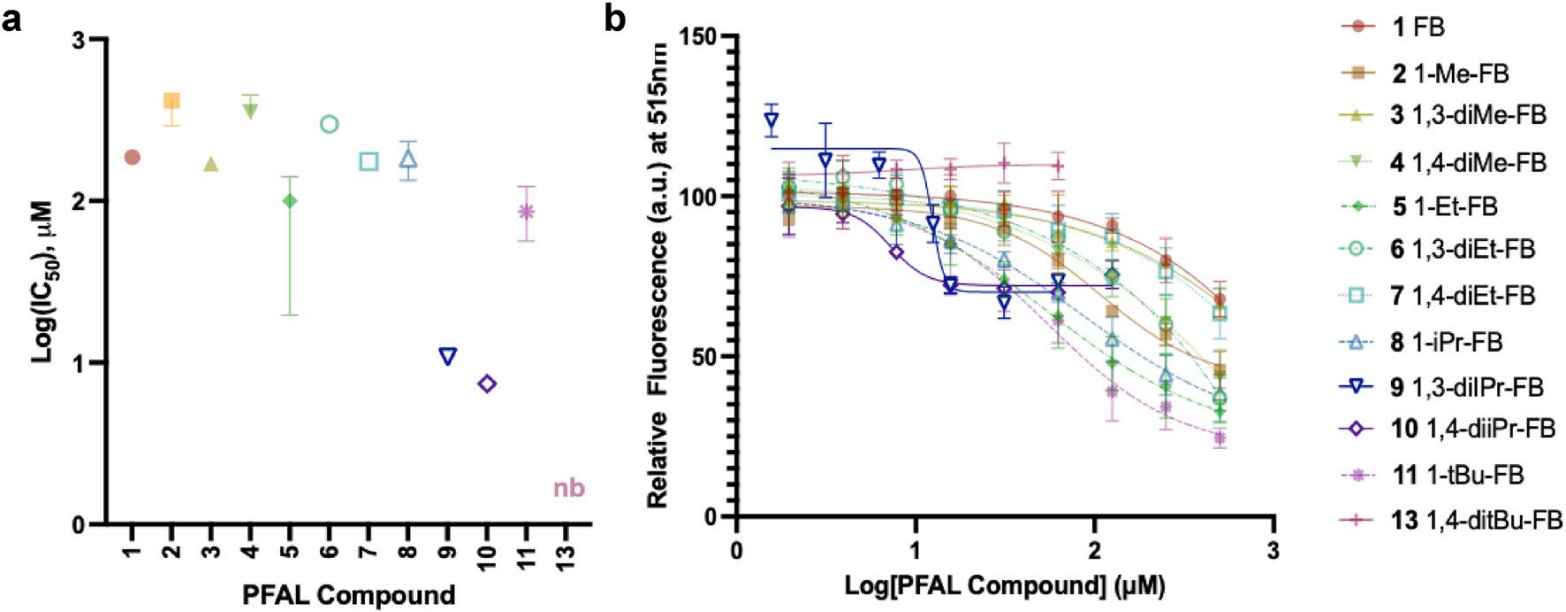
Binding of PFAL Compounds to Apoferritin. The relative binding affinity of PFAL compounds to HSAF (horse spleen apoferritin) was measured by displacement of the fluorophore 1-AMA (1-aminoanthracene) that demonstrates increased fluorescence when bound to HSAF. Error bars represent 95% CI of the calculated IC_50_, but upper limits were unable to be estimated for compounds **1**, **3**, **4**, **6**, or **7**. nb = non-binding (**a**) Calculated IC_50_ of PFAL compounds. (**b**) Binding curves from which IC_50_ values were calculated. Mean and standard deviation for each data point are shown.

The ability of anesthetics, to inhibit 1-aminoanthracene (1-AMA) binding to the ‘anesthetic site’ of model protein HSAF (horse spleen apoferritin) has been shown to correlate to anesthetic activity^22–24^. However, it is known that **9** also binds to this site^14^, suggesting the assay may also reveal optimal physicochemistry of other antagonists. Each compound in the library inhibited the binding of 1-AMA to HSAF (indicated by decreased fluorescence from unbound 1-AMA), except for compound **13** where testing was limited by solubility (Fig. 6). Calculation of IC_50_ values would suggest that compounds **9** and **10** are the most potent inhibitors of 1-AMA binding. However, the shape of these IC_50_ curves for **9** and **10** are markedly different from the other PFAL library members in that they demonstrate incomplete inhibition of 1-AMA binding which is similar to the binding seen by propofol in this system (Fig. S4). This similarity to propofol is consistent with the hypothesis of competitive antagonism of propofol. The appearance of incomplete competition at soluble concentrations is not explained by artifact of 1-AMA/PFAL interactions (Fig. S5) and is not readily explained by our current understanding of anesthetic binding in this system. Despite this, there is a similarity between the interactions of propofol, **9**, and **10** that differentiates the interactions of these compounds from the other PFAL analogues.

### Schild analysis

**Figure 7:**
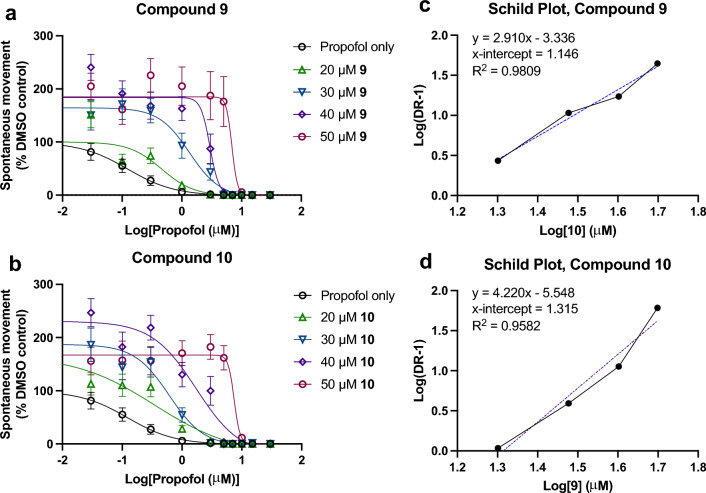
Schild Analysis of Propofol Antagonism with Compounds 9 and 10. (**a**,**b**) EC_50_ curves of propofol (SM) with co-administration of compounds 9 and 10 at 0, 20, 30, 40, and 50 μM. Error bars indicate 95% CI. (**c**,**d**) Schild plots derived from the EC_50_ data in sub-panels (**a**,**b**).

To begin an in vivo investigation of potential mechanisms of antagonism, a Schild analysis was conducted for both compounds **9** and **10**. EC_50_ curves for propofol with the addition of 0, 20, 30, 40, and 50 μM **9** and **10** were obtained (Fig. 7a-b), and then used to form the basis of Schild Plots (Fig. 7c-d). Compounds **9** and **10** exhibit overall linear correlations which is suggestive of competitive binding in a Schild analysis^25^; however, such a conclusion from this analysis would require all assumptions of a Schild analysis be met^26^, which is not the case in this system. A Schild analysis requires that an antagonist acts only at a single receptor type, but general anesthetics have many mechanistically relevant targets. Additionally, in a Schild plot for a competitive antagonist, a linear correlation of antagonism with a parallel rightward shift is seen across multiple orders of magnitude. This is in contrast to the plots found here for **9** and **10** that are linear across merely tens of micromolar concentrations (Fig. 7c-d) and do not show parallel rightward shifts (Fig. 7a-b) and thus cannot be said to be classical competitive antagonists. The slope of the linear fit is also > 1 for both compounds, which is often attributed to non-specific binding, which in some cases can be attributed to adsorption to glassware, but given the known non-specific binding of propofol in complex biological systems it is not surprising that these molecules could exhibit similar behavior to non-specific adsorption. Overall, this analysis must be viewed with caution as even if compounds **9** and **10** did act at a single site, propofol most likely does not which could explain the observation that these antagonists do not exhibit the “parallel shift” seen for a classical competitive antagonist. It is also important to note that these findings are observations of SM. A parallel analysis was conducted with observations of the mechanistically distinct EM, but no such relationship of antagonism was observed even with application of higher concentrations of **9** and **10** (see Fig. S6).

### Co-administration of compounds 9 and 10

**Figure 8:**
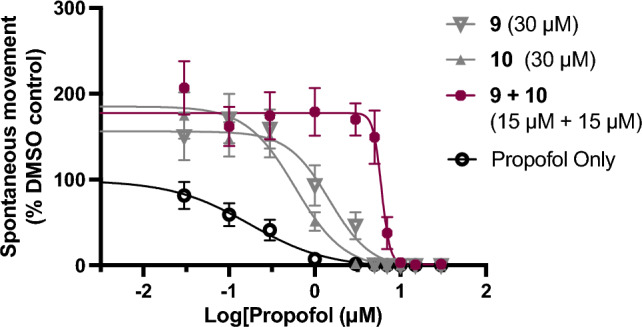
Synergism of compounds 9 and 10. To test for additivity, the antagonism of propofol by compounds 9 and 10 alone (30 μM) were compared to a combined dose of 9 and 10 (15 μM each) with a total PFAL concentration of 30 μM. Compared to 9 alone (EC_50_ = 1.3 μM), the combination of antagonists (9 + 10) at an equivalent concentration showed a threefold increase in antagonism (EC_50_ = 4.0 μM). Error bars indicate 95% CI.

To further examine the potential differences between compounds **9** and **10**, an additivity study was performed with both compounds co-administered with propofol at 15 μM each (for a total PFAL concentration of 30 μM). Compared to either compound administered alone there is an approximately threefold increase in EC_50_ indicating a synergism between these two compounds (Fig. 8). Given the complex and non-classic pharmacology of anesthetics, it is difficult to propose a specific mechanism of these antagonists from such an observation. Like anesthetics, these antagonists likely bind at multiple sites and multiple targets, but this synergism and the other data presented here support that these two lead compounds do not have identical mechanisms of action.

## Discussion

In this study, we have found that many fluorobenzene derivatives of propofol possess rapid antagonistic activity to propofol anesthesia in a larval zebrafish model by measures of spontaneous movement (SM). Differences in antagonist potency based on alkyl “side arms” exist and appear to correlate more to size/shape than solely their hydrophobicity as measured by LogP. Strikingly, no antagonism was seen via measures of elicited movement (EM), in addition to a lack of antagonism of two volatile anesthetics at clinically relevant concentrations, suggesting the antagonism of SM by these alkyl-flourobenzene molecules is more specific to propofol and not due solely to non-specific CNS stimulation.

Like the mechanism(s) of anesthesia generally, the mechanism(s) of antagonism by these alkyl-fluorobenzenes remains unknown. It would be logical to propose that molecules that are structurally similar to propofol, would act by competitive antagonism, but at what target(s)? Anesthetics are known to be ‘promiscuous’, interacting with many binding partners, and thus the possible targets or subset of anesthetic targets mediating this effect could be quite large in number. The large number of potential targets contributing to anesthetic endpoints may also be why these compounds appear to be ‘surmountable’ antagonists if they are only antagonizing some subset of targets. Despite increasing concentrations of **9**/**10**, there is consistently zero movement observed by approximately 10 μM propofol and a corresponding increase in slope with increased PFAL concentration (see Fig. 7a-b). The GABA_A_R is most prominently implicated in anesthetic (esp. propofol) mechanisms^27^, but compound **9** has previously been shown to produce no significant modulation of a propofol-sensitive GABA_A_R, and thus these findings are not likely to be GABA_A_R mediated^15^. While the linearity of the Schild plots might be superficially interpreted as competitive antagonism, consistent with the hypothesis based on structural and physicocochemical similarity, there are important limitations to a Schild analysis that are not met by this system which means that these compounds cannot be said to be competitive antagonists despite other evidence that points to behavior similar to propofol.

Because of the absence of validated, easily accessible in-vivo molecular targets, surrogate targets have often been employed to study anesthetic protein binding. Principal among them have been firefly luciferase^28^ and apoferritin^29,30^, both of which have crystal structures with bound anesthetic. However, they are less than ideal models because each bind compounds that do not produce anesthesia, as shown here with apoferritin. However, it is curious that antagonistic compounds **9** and **10** have similar binding curves as propofol (Fig. S4) where a calculated EC_50_ values show higher affinity in part due to incomplete competitive binding. In a screen for anesthetics using HSAF as a model for anesthetic binding, one might erroneously conclude that **9** and **10** would be anesthetics^23,24^. However, the similarities in binding of **9**, **10**, and propofol suggest they interact with HSAF in a similar manner that is different from the other PFAL compounds that were able to compete with 1-AMA (albeit with a lower binding affinity), but are neither anesthetics nor effective antagonists. These results suggest that binding to HSAF without further interpretation is not an ideal anesthetic model but it does provide a physicochemical model that seems to distinguish propofol and its antagonists from other less active molecules.

Anesthetic agents across many species are known to have steep population dose–response curves^31–33^, so it is not unreasonable to expect an anesthetic antagonist to have a similar features. The basis for these steep dose response relationships is debated, but one explanation relies on complexity, or the number of contributing molecular targets^27,31,33,34^. Certainly, the most potent antagonist, **10**, appears to possess this feature, with a very steep dose response relationship. The more gradual change in antagonistic effect of **9** is typical of more specific pharmacologic agents. The steep slope observed with the EC_50_ curve of **10** (Fig. 5d) is likely indicative of differences in interactions between **9** and **10** despite their seemingly subtle differences in carbon-backbone. This slope is so steep, the flip between anesthetized states happens like a ‘switch’, which is not characteristic of competitive antagonism, and thus also supports potential mechanistic differences brought about by the subtle structural changes between these compounds. What is more surprising is the apparent synergism observed with co-administration of **9** and **10**. Differences in mechanism are not only potentially interesting for further study of antagonists, but piecing together the underpinnings of antagonist function could lay the groundwork for further understanding of molecular mechanisms of anesthetics, and potentially novel druggable targets. Efforts to elucidate the yet unidentified targets for both propofol and these PFAL compounds, is an important area of future investigation. Nevertheless, even without a clear mechanism, the ability to reverse propofol-induced anesthesia could prove to be an important control in studies from the molecular to network levels, and like the ‘non-immobilizers’^35^, adds to the potential toolbox for understanding anesthetic actions.

The alkyl-fluorobenzene molecules tested in this study are just a small sampling of the vast number of possible configurations of an alkylphenol or alkyl-fluorobenzene. Recent work on ciprofol highlights how even a seemingly small number of carbon arrangements and chirality centers can become both increasingly complicated, but have a significant effect on potency^36^. However, a potentially more interesting avenue of further study might be to instead alter electronic configurations to further elucidate the key features differentiating anesthetics and antagonists or even other “inactive” molecules with yet uncharacterized biologic effects. By augmenting or diminishing certain features of propofol, it may be possible to fine tune the functionality of these molecules that interact with likely many untold targets.

A principal limitation one might posit in this study is the non-mammalian model used to determine activity. However, the larval zebrafish model is a vertebrate that is phylogenetically closer to mammals than other anesthetic models such as flies or worms. Zebrafish also exhibit complex behaviors, and have demonstrated learning, and their anesthetic sensitivity has been shown to correlate to mammals^17,37–39^. Nonetheless, the zebrafish is a genetically heterogeneous model, and thus can have wider variation in their responses to a given condition compared to rodent models. This variation provides a more stringent test of experimental conditions and more closely mimics the phenotypic diversity seen in humans. Additionally, the larvae are still developing, and like mammals, anesthetic potency changes as a function of age. Thus, the consistency of these findings in other developmental stages, and the translation of these results to rodent models are important areas of future investigation.

In summary, we describe an innovative approach to terminating anesthetic activity, and provide a screen of alkyl-fluorobenzenes as one potential propofol antagonist chemotype. In the larval zebrafish model, we found that these compounds are antagonistic to propofol-induced immobility. Further, even the most potent propofol antagonists do not antagonize volatile anesthetic-induced immobility. This finding along with the initial pharmacologic investigations presented here, do not support a model of classical competitive antagonism. These findings do not entirely rule out some type of competitive interaction at one of the many propofol binding sites, but these compounds may also simply be physiologic antagonists. With current information, precise classification of these antagonists remains indeterminant. Like anesthesia itself, mechanisms of antagonism are yet to be revealed, but are unlikely to be as simple as competitive binding at a single site. This is likely only a small subset of molecules that antagonize propofol anesthesia, and additional improvements in potency will further aid in mechanistic investigations that underpin these curious findings.

## Methods

### Library compounds

All compounds were purchased from commercial sources including ‘make on demand’ custom synthesis via ChemSpace: **1**: Sigma-Aldrich, F6001; **2**: Chem Space; **3**: Aldrich, 452,866; **4**: Chem Space; **5**: Chem Space; **6**: Sigma-Aldrich, S567299; **7**: Chem Space; **8**: Chem Space; **9**: synthesized as previously published^15^ **10**: Chem Space; **11**: Chem Space; **13**: Chem Space. For Characterization data of compounds purchased from Chem Space, see Figs. S7–S22. Compound **6** was purchased with no guarantee of identity or purity and was thus characterized after purchase (See NMR and LC in Figs. S23, S24). Physiochemical properties were calculated with the Molinspiration property calculation toolkit (Molinspiration Chemoinformatics). LogP values were calculated with Chem Draw 21.0.0.

### Zebrafish husbandry

All zebrafish experiments were conducted in accordance with approval by the University of Pennsylvania Institutional Animal Care and Use Committee (IACUC) and performed according to ARRIVE guidelines. Adult animals were housed and maintained at the University of Pennsylvania’s aquatic facility, and larvae were reared in a satellite facility, both of which are overseen by the University Laboratory Animal Resources. Adult mating pairs of Tübingen long fin (TLF) zebrafish were routinely cycled to ensure biological diversity of clutches. Fish were kept on a 14-h light/10-h dark cycle at 28 °C in E3 embryo water (5 mM NaCl, 0.17 mM KCI, 0.33 mM CaCl_2_, 0.33 mM MgSO_4_, pH 7.4) until 5 days post fertilization (dpf). After experiments, euthanasia by rapid cooling was conducted in accordance with IACUC protocols.

### Zebrafish behavior and compound toxicity

Behavioral experiments (screening, EC_50_ curves) were performed as previously reported^17^. Antagonism after induction was conducted in a similar manner with different timepoints and solution changes as indicated. The DanioVision Observation Chamber (Noldus) and Ethovision XT16 software were used to track and record (1280 × 960 resolution at 25 frames per second) larval zebrafish (5 dpf) movement in glass 96-well plates (Zissner North America 3,600,500). All experiments were performed at 28 °C with the DanioVision Temperature Control Unit. Zebrafish were allowed to equilibrate in E3 and 2% DMSO (with or without drug) for 10 min before being placed in the observation chamber for 22 min of recording. Two different endpoints were used to assess depth of anesthesia: spontaneous movement (SM) and elicited movement (EM). In these measures, fish movement was considered to be non-anesthetized. During each observation, fish were given time to acclimate to the chamber, and minutes 15–19 of the recording were used for analysis of SM. An EM response using the Daniovision tapping device (8 out of 8 intensity, movement response measured for 1 s after stimulus) was also measured at the end of the SM observation period. All recordings were scrutinized by a blind observer for technical errors in tracking to ensure accuracy of zebrafish detection and inaccurate tracking was excluded from further analysis. Toxicity was measured in separate experiments by exposing larvae to compounds for 30 min, transferring to fresh E3, and then observing mortality after exposure. LD_50_ values were calculated from 24-h survival.

### Drug preparation and administration

All concentrated stocks of propofol (Sigma-Aldrich D126608) and library compounds were made in DMSO (Fisher BP231-1) and stored at −20 °C. Final concentration of solutions for administration to fish were in E3 with 2% DMSO. Special care was taken in preparation of the sevoflurane (AbbVie Inc.) and isoflurane (Piramal Critical Care) solutions given their high vapor pressures (see Supplemental Methods and Figs. S25, S26). Due to the hydrophobic nature of all library compounds and anesthetics tested, all solutions were stored in glass vials and glass 96-well plates were used for all behavioral experiments.

### Statistical analysis

All experiments were replicated with either three technical replicates or at least three biologic replicates (separate clutches on separate days with 12 fish per condition per replicate). For most experiments, 5 biologic replicates were conducted generating 50–60 data points from individual fish per condition. All statistical analysis was performed with GraphPad Prism version 10.1.1. ROUT test for outliers were performed with the default Q value of 0.1%, RAW-One-way ANOVA with Sidak's multiple comparison test. All EC_50_ and IC_50_ curves were fitted in Prism with the model Absolute EC_50_, X is log(concentration) with baseline constraint equal to zero.

### 1-Aminoanthracene (1-AMA) competition fluorescence assay

The assay was performed as previously described^16^. See Supplemental Methods for further detail.

### Supplementary Information

Supplementary Information 1.

Supplementary Video 1.

## Data Availability

The datasets generated during and/or analyzed during the current study are available from the corresponding author on reasonable request.
