# Supplementary material for: Antagonism of propofol anesthesia by alkyl-fluorobenzene derivatives
Source: Sci Rep. 2024 Jul 10;14:15943. doi: 10.1038/s41598-024-66672-z (PMC11236999; doi:10.1038/s41598-024-66672-z)
Supplement: Supplementary file 1 — Supplementary Information 1. [file 41598_2024_66672_MOESM1_ESM.pdf]

Antagonism of Propofol Anesthesia by Alkyl-fluorobenzene Derivatives

Diana M. Plasencia, Liam H. Rodgers, Alexys R. Knighton,
Roderic G. Eckenhoff, E. Railey White

University of Pennsylvania, Department of Anesthesiology & Critical Care,
Philadelphia, United States of America

<u>Supplemental Table of Contents</u> <u>Page</u>

Supplemental Methods		2
Supplemental References		3
Table S1	Physicochemical Properties of PFAL Compounds	3
Figure S1	Toxicity of PFAL Compounds Alone and with Propofol	4
Figure S2	Spontaneous Movement Tracings of 5pdf Zebrafish Exposed to PFAL Compounds	5
Figure S3	Estimated Propofol EC_{50} Values	6
Figure S4	Binding of Propofol and Compound **9** to Apoferritin/HSAF	7
Figure S5	Fluorescence of 1-AMA in the presence of Compound **9**	7
Figure S6	Elicited Movement Propofol E_{50} Curves at various concentrations of Compounds **9** and **10**	8
Figure S7	^{1}H NMR (400 MHz) of compound **2** (1-methyl-2-fluorobenzene)	9
Figure S8	GC/MS of compound **2** (1-methyl-2-fluorobenzene)	10
Figure S9	^{1}H NMR (400 MHz) of compound **4** (1,4-dimethyl-2-fluorobenzene)	11
Figure S10	GC/MS of compound **4** (1,4-dimethyl-2-fluorobenzene)	12
Figure S11	^{1}H NMR (400 MHz) of compound **5** (1-ethyl-2-fluorobenzene	13
Figure S12	GC/MS of compound **5** (1-ethyl-2-fluorobenzene)	14
Figure S13	^{1}H NMR (400 MHz) of compound **7** (1,4-diethyl-2-fluorobenzene)	15
Figure S14	GC/MS of compound **7** (1,4-diethyl-2-fluorobenzene)	16
Figure S15	^{1}H NMR (400 MHz) of compound **8** (1-isopropyl-2-fluorobenzene)	17
Figure S16	GC/MS of compound **8** (1-isopropyl-2-fluorobenzene)	18
Figure S17	^{1}H NMR (400 MHz) of compound **10** (1,4-diisopropylfluorbenzene)	19
Figure S18	GC/MS of Compound **10** (1,4-diisopropylfluorbenzene)	20
Figure S19	^{1}H NMR (400 MHz) of compound **11** (1-tertbutyl-2-fluorobenzene)	21
Figure S20	HPLC of compound **11** (1-tertbutyl-2-fluorobenzene)	22
Figure S21	^{1}H NMR (400 MHz) of compound **13** (1,4-ditertbutyl-2-fluorobenzene)	23
Figure S22	GC/MS of compound **13** (1,4-ditertbutyl-2-fluorobenzene)	24
Figure S23	^{1}H NMR (400MHz) of Compound **6** (1,3-diethyl-2-fluorobenzene)	25
Figure S24	HPLC of compound **6** (1,3-diethyl-2-fluorobenzene)	26
Figure S25	Effect of 3M Adhesive on 5 dpf Zebrafish Movement	27
Figure S26	Concentration of Sevoflurane and Isoflurane in the Glass 96-well Plates Over Time	27

Supplemental Methods:

Behavioral Experiments with Volatile Anesthetics. Solutions of inhaled volatile anesthetics were stored in glass vials with minimal head space and were transferred utilizing Hamilton gas-tight syringes to prevent evaporative loss. Isoflurane (Primal Care) was stored at 4°C, and sevoflurane (AbbVie Inc.) was stored at -20°C. Solutions were made in DMSO and then diluted in glass scintillation vials to the desired experimental concentrations in E3 (embryo water). Each vial contained 22 mL of E3 which minimized head space to prevent drug loss. Vials were sealed quickly after addition of volatile anesthetics to minimize loss. Solutions were mixed via vortexing, and anesthetics dissolved readily in the final DMSO concentration of 2%. For behavior experiments the zebrafish were placed in the 96 well glass plate (Zissner North America 3600500), and E3 solutions containing drug were quickly pipetted into each well, with slight over filling to prevent entrapment of air bubbles during application of adhesive strips (3M 300LSE). The zebrafish were allowed to equilibrate in the solution for 10 minutes prior to observation for 22 minutes in the behavior system. Glass 96-well plates were inverted (adhesive side down) when placed in the behavior system. As shown in Fig. S25, the presence of the adhesive did not have a significant change in zebrafish behavior. To assure consistent concentration of volatile anesthetic, this experiment was repeated without fish and concentrations were measured by HPLC. See methods below and Fig. S26.

High Performance Liquid Chromatography (HPLC) of Volatile Anesthetics in Glass 96-well Plates. The methodology used for behavior experiments was repeated to prepare the substances for high pressure liquid chromatography. The drug was then removed from the wells with a Hamilton Gas-Tight syringe (without taking the adhesive strip off) and dispensed into a glass HPLC vial, that were tightly sealed and wrapped in parafilm. The concentration of volatile anesthetic was measure in multiple wells at time points from 15-180 minutes as compared to a standard curve of each anesthetic. Analysis of HPLC occurred immediately after each time point (15-minute increments). Standard solutions of volatile anesthetic in methanol were freshly prepared prior to each experiment. Samples were injected into the Beckman Coulter HPLC system equipped with an analytical Eclipse Plus C18 column (Agilent Technologies) and Waters 2410 refractive index detector. The mobile phase was a single solution of acetonitrile and 20 mM phosphate buffer (pH 4) at 23:77 v/v at a flow rate of 1.0 mL/min. Chromatic peaks for the substances were identified by retention time and compared to standard curves.

1-Aminoanthracene Competition Fluorescence Assay. This assay was performed in a similar manner to that previously described with a few modifications.[4,5] A saturated solution of 1-AMA (1-aminoanthracene, Sigma-Aldrich) was prepared by sonication in a 1x phosphate buffered saline (PBS, pH = 7.4) followed by filtration with a 0.22 μm polyvinylidene fluoride filter (PVDF, Rephile). After filtration, the 1-AMA concentration was determined by UV−vis spectroscopy utilizing an experimentally determined extinction coefficient of 1-AMA (Σ_{368} = 4073 M^{-1} cm^{-1}).[4] Stock solutions of ligand were prepared in DMSO. Horse spleen apoferritin (HSAF, Sigma-Aldrich, 0.2 μm filtered) was used as received. 1-AMA (15 μM assay concentration) was preequilibrated with HSAF (15 μM assay concentration of dimer), and 5 μL of ligand stock in DMSO was then added for a final volume of 500 μL (1% DMSO v/v in PBS). Ligand concentrations ranged from 1 to 125 μM except where solubility limited the upper range. Upon addition of the ligand, the samples were mixed and immediately analyzed with a spectrofluorometer (Shimadzu RF-5301 PC) with an excitation wavelength of 380 nm and emission detection from 400 to 700 nm. The fluorescence curves were corrected by subtracting contributions from 1-AMA alone and HSAF alone. Fluorescence intensities at 515 nm were plotted as a percentage of the control (1-AMA and HSAF bound with no competing ligand present) and were fitted to a logarithmic four parameter variable slope using GraphPad Prism (version 9.4.0, GraphPad Software, La Jolla California, USA). The result of these experiments can be seen in Figure 4 in the main text.

Supplemental References

(1) Kelz, M. B.; Sun, Y.; Chen, J.; et al. An Essential Role for Orexins in Emergence from General Anesthesia. *Proc. Natl. Acad. Sci. U. S. A.* **2008**, *105* (4), 1309–1314.

(2) Friedman, E. B.; Sun, Y.; Moore, J. T.; et al. A Conserved Behavioral State Barrier Impedes Transitions between Anesthetic-Induced Unconsciousness and Wakefulness: Evidence for Neural Inertia. *PLoS One* **2010**, *5* (7).

(3) Wei, H.; Kang, B.; Wei, W.; et al. Isoflurane and Sevoflurane Affect Cell Survival and BCL-2/BAX Ratio Differently. *Brain Res.* **2005**, *1037* (1–2), 139–147.

(4) White, E. R.; Leace, D. M.; Bedell, V. M.; et al. Synthesis and Characterization of a Diazirine-Based Photolabel of the Nonanesthetic Fropofol. *ACS Chem. Neurosci.* **2021**, *12* (1), 176–183.

(5) Butts, C. A.; Xi, J.; Brannigan, G.; et al. Identification of a Fluorescent General Anesthetic, 1-Aminoanthracene. *Proc. Natl. Acad. Sci.* **2009**, *106* (16), 6501–6506.

Table S1. Physicochemical Properties of PFAL Compounds.

	MW (g/mol)	MV (Å)	cLogP
Propofol	178.28	191.96	3.70
**1**	96.10	88.97	2.10
**2**	110.13	105.54	2.50
**3**	124.16	122.10	2.90
**4**	124.16	122.10	2.93
**5**	124.16	122.34	2.97
**6**	152.21	155.70	3.83
**7**	152.21	155.70	3.86
**8**	138.16	138.19	3.09
**9**	180.27	188.87	4.08
**10**	180.27	188.97	4.58
**11**	152.21	155.16	3.76
**13**	208.32	221.35	5.44

cLogP = calculated octanol/water partition coefficient
MW = molecular wegith
MV = molecular volume

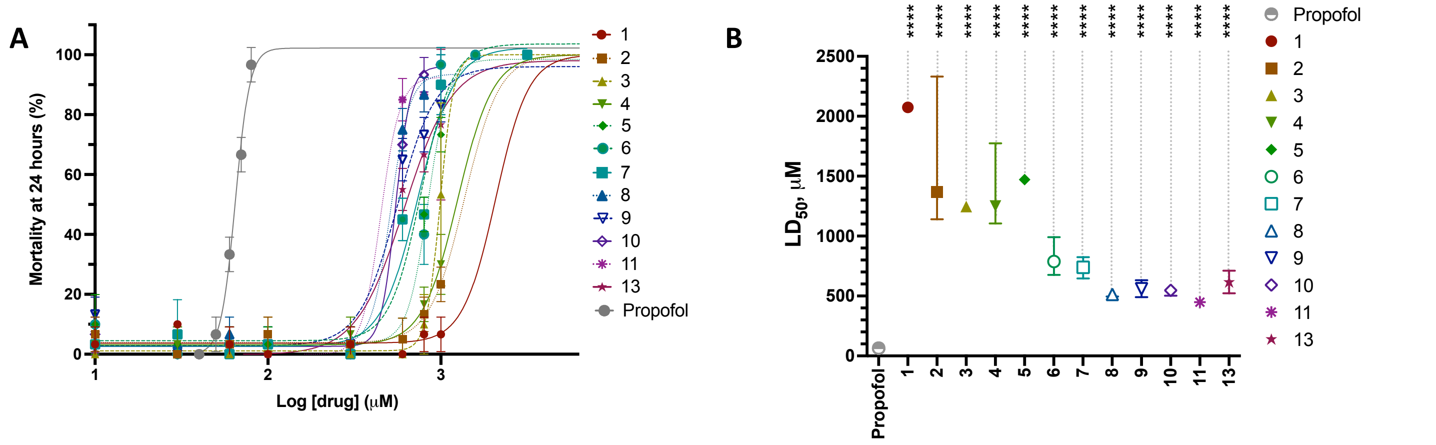

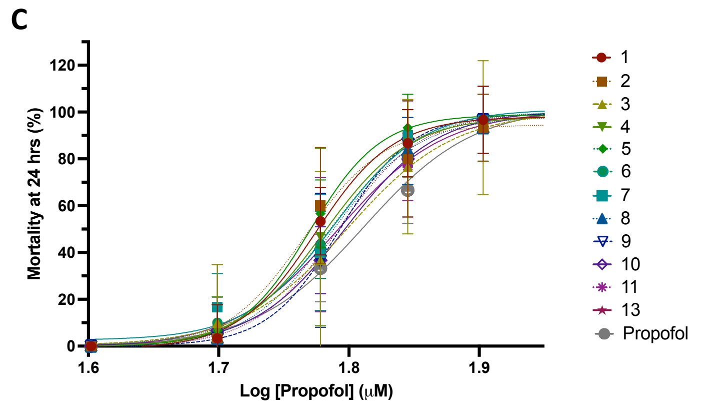

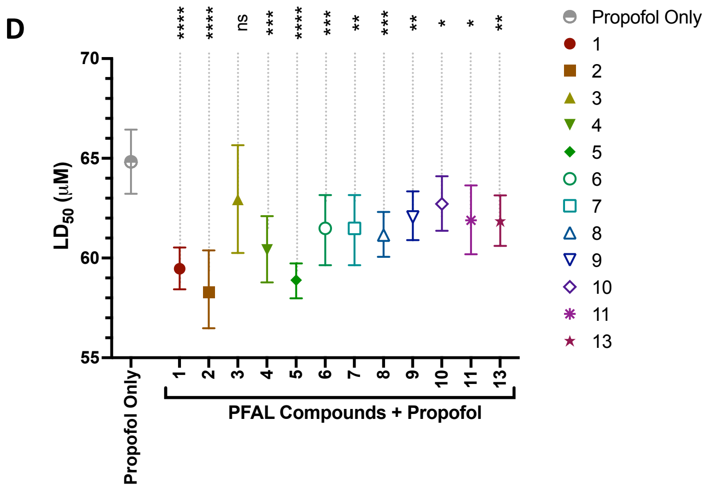

Figure S1: Toxicity of PFAL Compounds Alone and with Propofol. A) Toxicity of Propofol only and each PFAL compound alone (no propofol), with each PFAL compound showing much less toxicity than Propofol. Mean and standard deviation are shown for each data point. B) Comparison of LD_{50} values from Panel A. LD_{50} and 95% CI are shown for each data point. Due to limitations in PFAL solubility and the resultant limitations in data collection, 95% CI was unable to be determined for **1** and **11** and no upper limit of 95% CI was determined for **8** and **10**. C) To further characterize the toxicity of the PFAL compounds, they were co-administered at 30 μM compound with variable concentrations propofol. This showed a small increase in toxicity compared to administration of propofol alone. D) Comparison of LD_{50} values from panel C. Comparison of LD_{50} values from Panel C. 95% CI are shown for each data point. E) Calculated LD_{50}s from Panel A and C. ns: not significant, P > 0.05, *: P ≤ 0.05, **: P ≤ 0.01, ***: P ≤ 0.001, ****: P ≤ 0.0001

E

Compound	LD_{50}, Drug Alone (μM, 95% CI)	LD_{50}, co-administration with propofol (μM, 95% CI)	P-value (comparison to Propofol LD_{50})
Propofol only	65 (63 to 67)	---	---
1	2074 (???)	59 (58-60)	<0.0001
2	1369 (1141 to 2331)	58 (56-60)	<0.0001
3	990 (938 to 1056)	63 (60-66)	0.2149
4	1250 (1105 to 1774)	60 (59-62)	0.0005
5	845 (800 to 892)	59 (58-60)	<0.0001
6	769 (673 to 852)	61 (60-32)	0.0007
7	736 (646 to 824)	61 (60-63)	0.0057
8	513 (459 to ???)	61 (60-62)	0.0004
9	560 (491 tp 631)	62 (61-63)	0.0081
10	547 (503 to ???)	63 (61-64)	0.0425
11	449 (???)	62 (60-64)	0.0135
13	616 (522 to 712)	62 (61-63)	0.0044

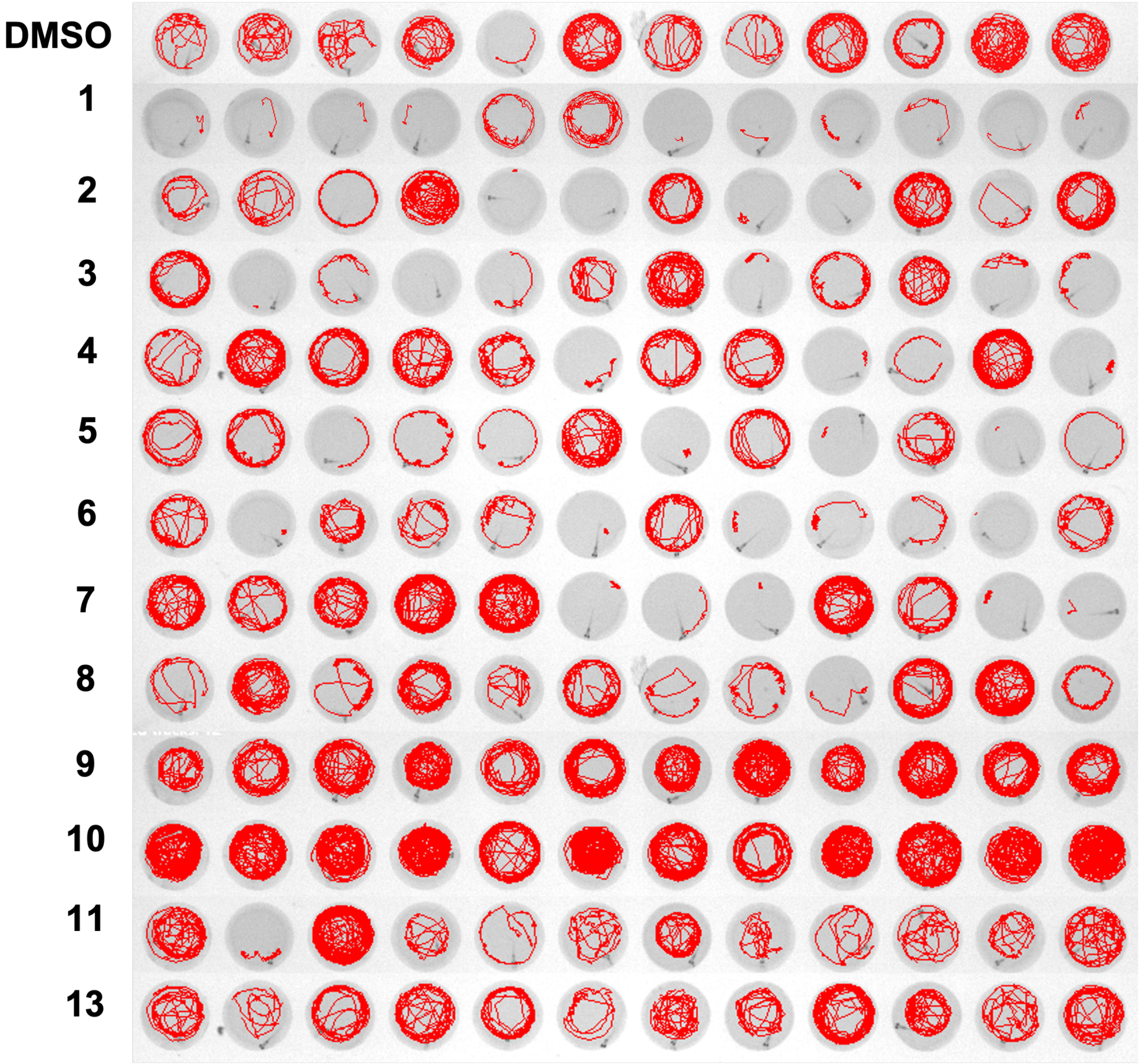

Figure S2. Spontaneous Movement Tracings of 5pdf Zebrafish Exposed to PFAL Compounds. Tracings (red lines) of spontaneous movement are shown from one row of a 96-well plate from a single biologic replicate where 5 dpf zebrafish were exposed to 30 μM PFAL compounds (or DMSO control). Each well has a total DMSO concentration of 2%. Tracings are shown for spontaneous movement during minutes 14-19 of the observation period. Video of fish movement can be seen in the supplemental video.

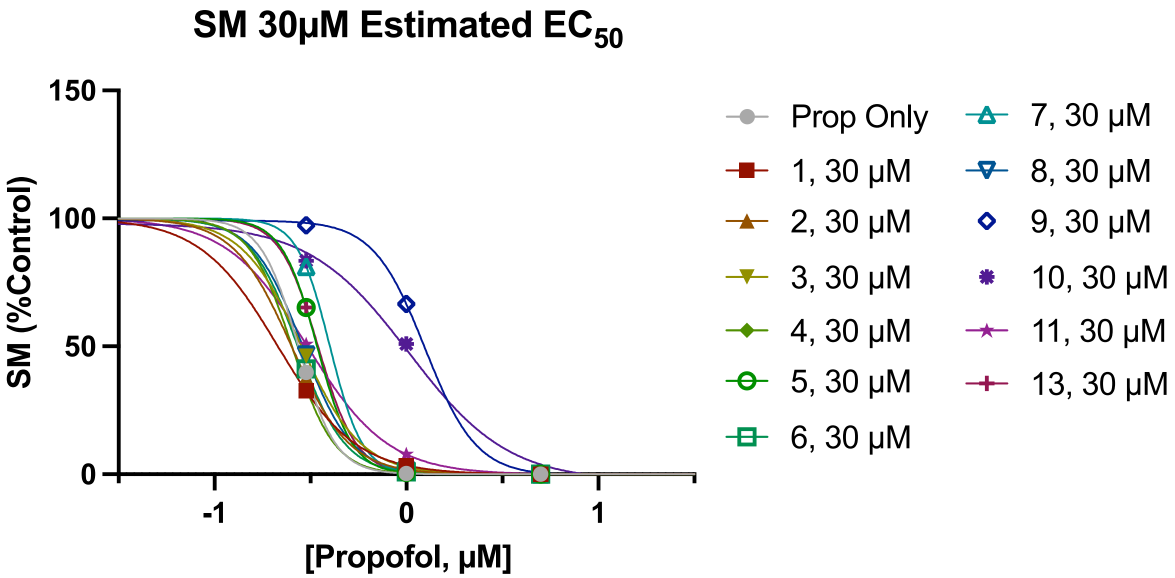

Figure S3. Estimated Propofol EC_{50} Values. From the screening of PFAL Compounds (data presented in Fig. 2 from the main text), estimated EC_{50} values for spontaneous movement (SM) were calculated (with upper and lower constraints at 100 and 0 respectively). More formal EC_{50} values were determined for the the most potent compounds (9 and 10). This data can be seen in the main text (Fig. 6).

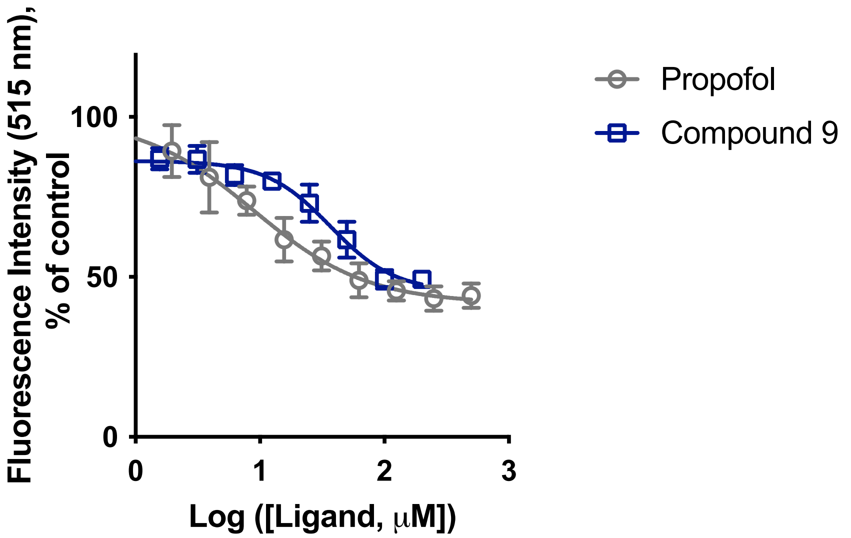

Figure S4. Binding of Propofol and Compound 9 to Apoferritin/HSAF. In the same 1-AMA/HSAF assay seen in Figure 4 in the main text, this graph shows the similarity in the binding curves of propofol and compound 9.

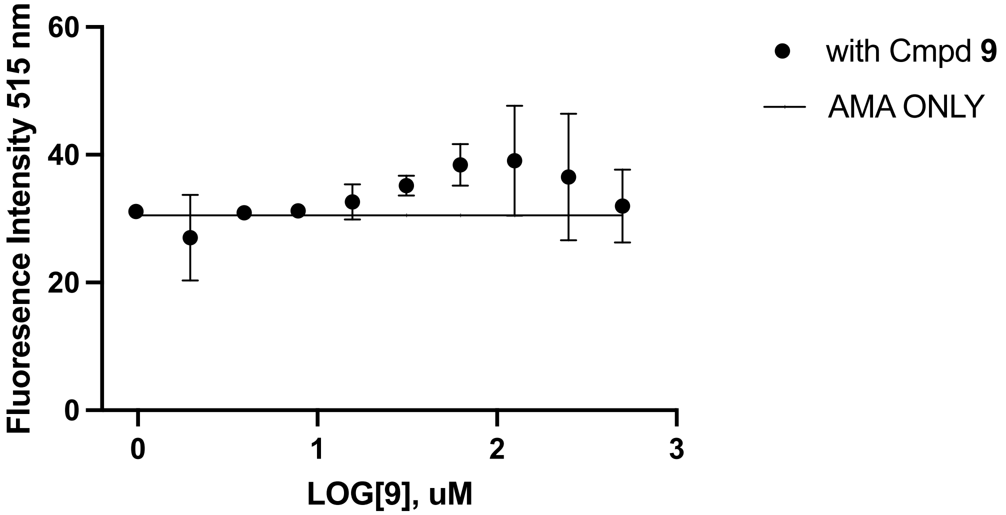

Figure S5. Fluorescence of 1-AMA in the presence of Compound 9. To test for fluorescent interference by a library compound via inner filter effect or other mechanism, a control study was done with compound and 1-AMA alone (no apoferritin/HSAF is present in these samples). At concentrations higher than those used in the 1-AMA assay for compound 9 (see Figure 4 in the main text), some enhancement of fluorescence was found, but this would not account for the differences in the binding/displacement curves seen with PFAL members.

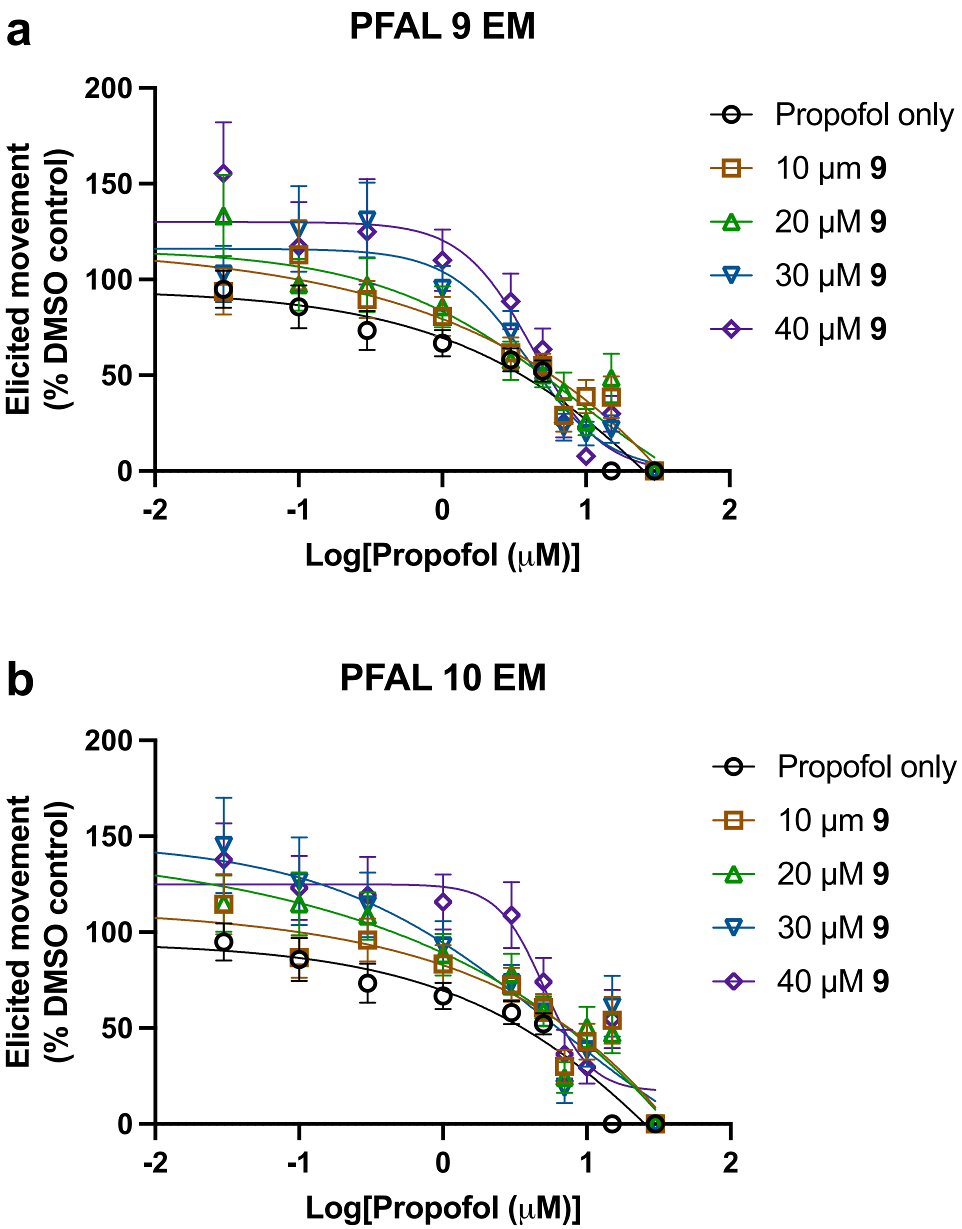

Figure S6. Elicited Movement Propofol E_{50} Curves at various concentrations of Compounds 9 and 10. a and b) EC_{50} curves of propofol (EM) with co-administration of compounds **9** and **10** at 0, 20, 30, and 40 μM. Error bars indicate 95% CI.

Figure S7. ^{1}H NMR (400 MHz) of compound **2** (1-methyl-2-fluorobenzene)

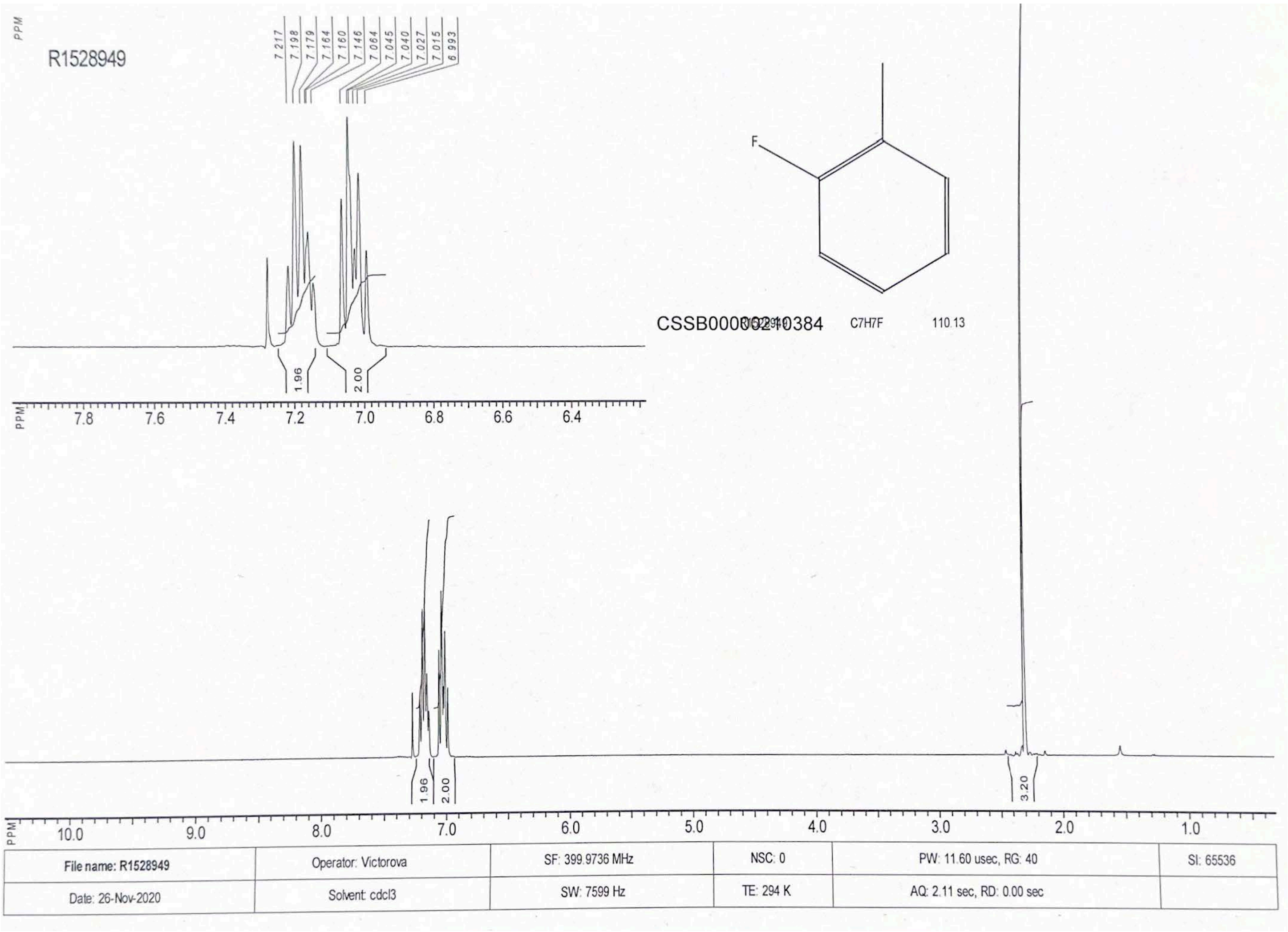

^{1}H NMR 400 MHz $CDCl_3$ δ 2.3 (s, 3H), 6.99-7.06 (m, 2H), 7.15-7.22 (m, 2H).

Figure S8. GC/MS of compound **2** (1-methyl-2-fluorobenzene)

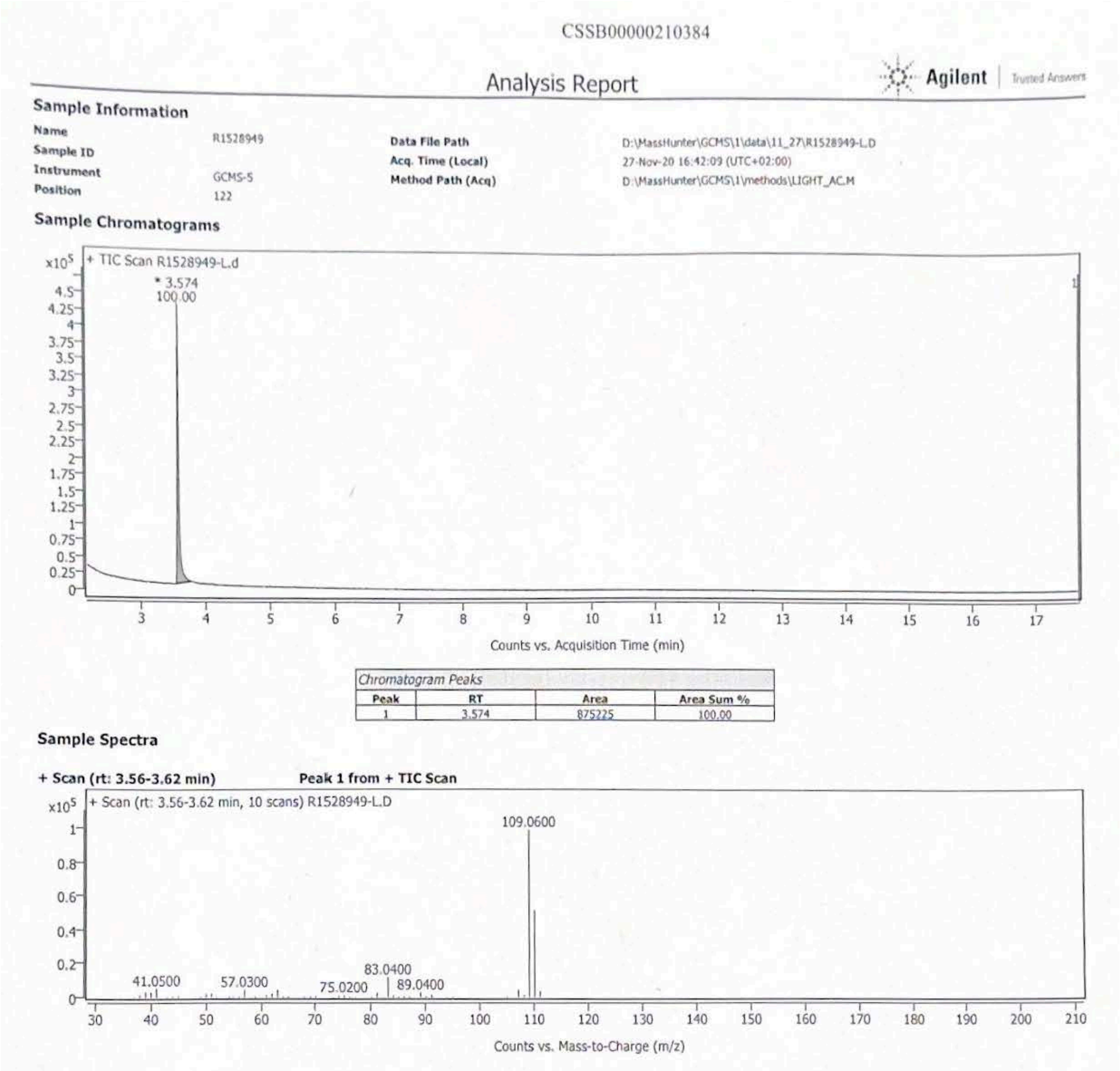

Figure S9. ^{1}H NMR (400 MHz) of compound **4** (1,4-dimethyl-2-fluorobenzene)

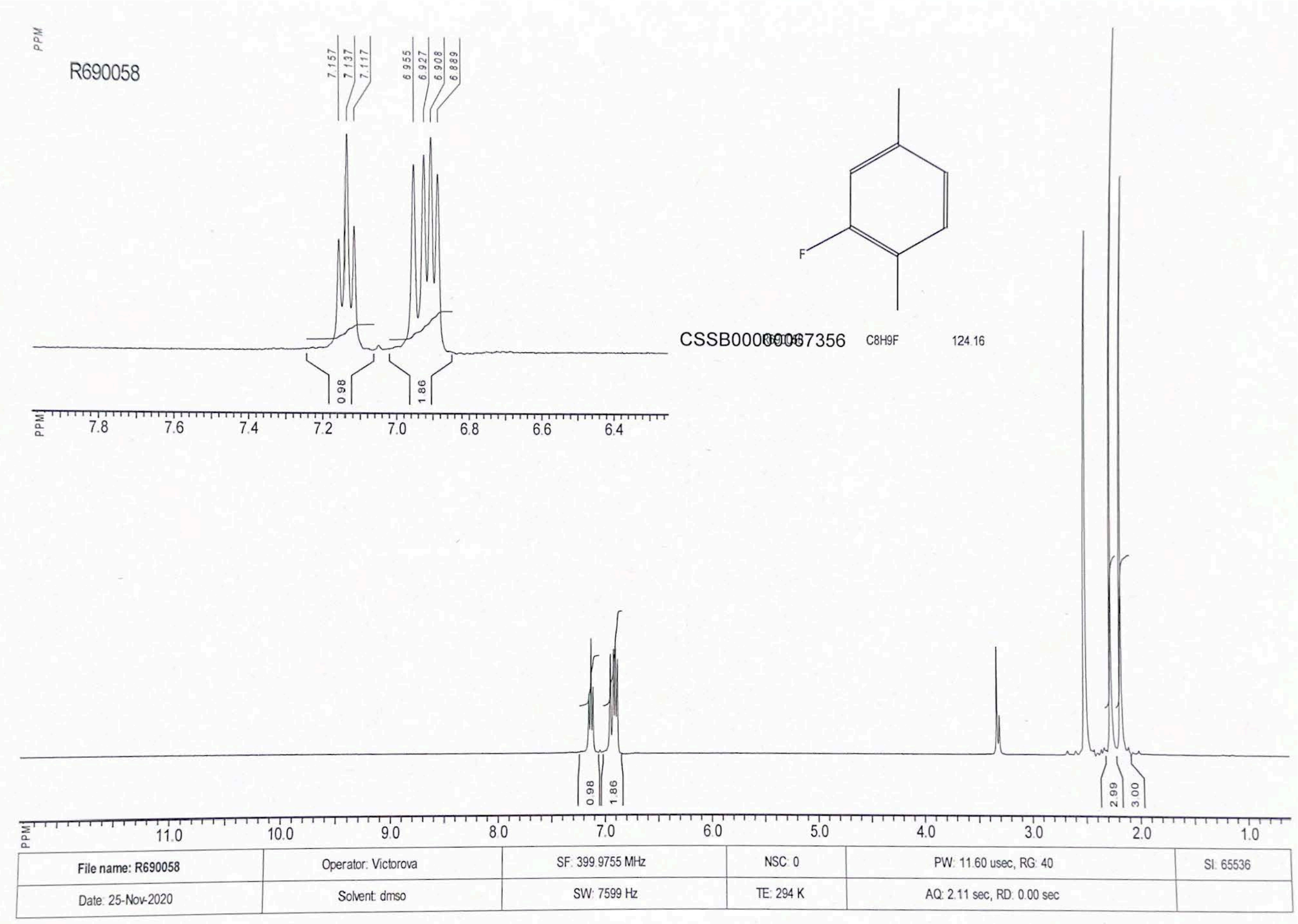

^{1}H NMR 400 MHz DMSO δ 2.2 (s, 3H), 2.3 (s, 3H), 6.89-6.93 (t, 1H), 6.96 (s, 1H), 7.12-7.16 (t, 1H).

Figure S10. GC/MS of compound **4** (1,4-dimethyl-2-fluorobenzene)

```
Data Path : C:\msdchem\1\data\06_23\
Data File : R690058.D
Acq On    : 23 Jun 2016  13:24
Operator  :
Sample    : R690058
Misc      : CH3OH
ALS Vial  : 4   Sample Multiplier: 1

Search Libraries:   C:\Database\EMPTY.L              Minimum Quality:   0

Unknown Spectrum:   Apex
Integration Events: ChemStation Integrator - autoint1.e
```

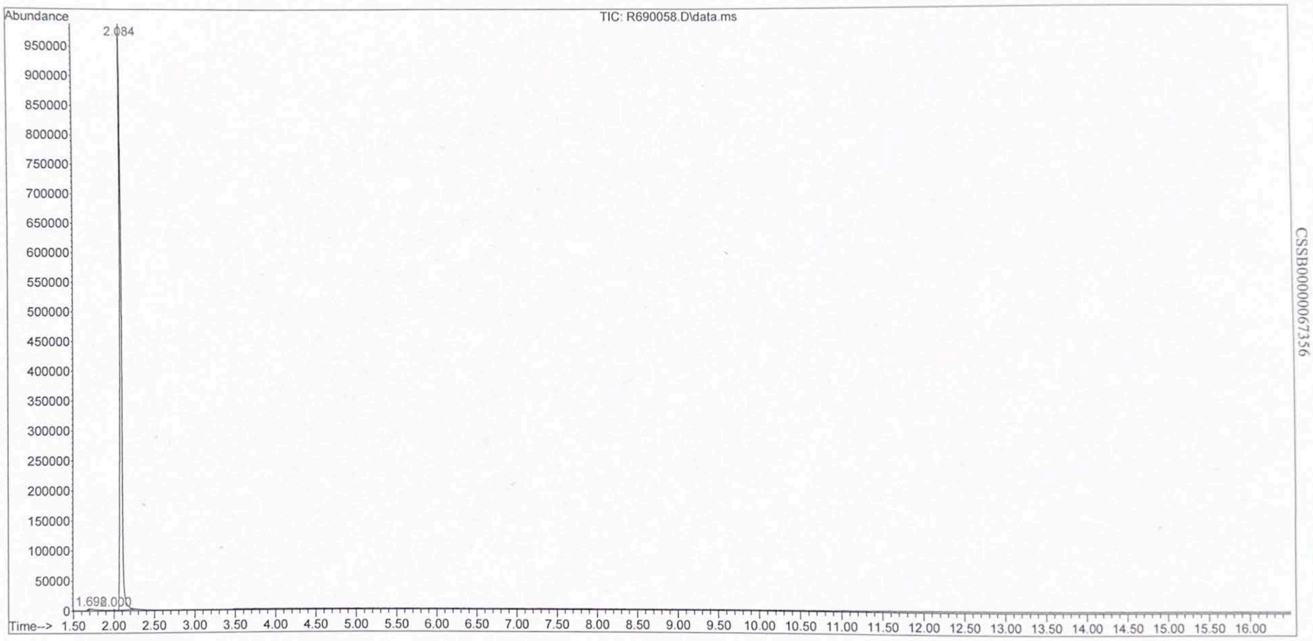

Unknown Spectrum based on Apex

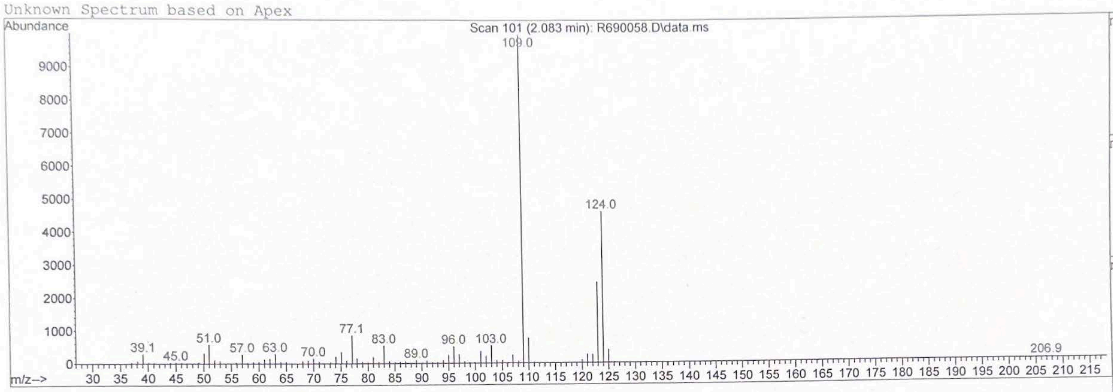

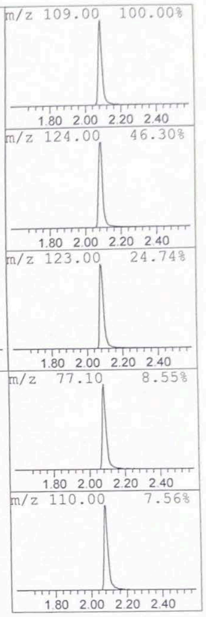


```
Data File: C:\msdchem\1\data\06_23\R690058.D
Sample   : R690058

Peak Number:  3     at      2.083 min  Area:  15205124  Area % 99.68

 The 3 best hits from each library.          Ref\#     CAS\#       Qual
--------------------------------------------------------------------
    C:\Database\EMPTY.L      No hits were retrieved.
```

CSSB00000067356

Figure S11. ^{1}H NMR (400 MHz) of compound **5** (1-ethyl-2-fluorobenzene)

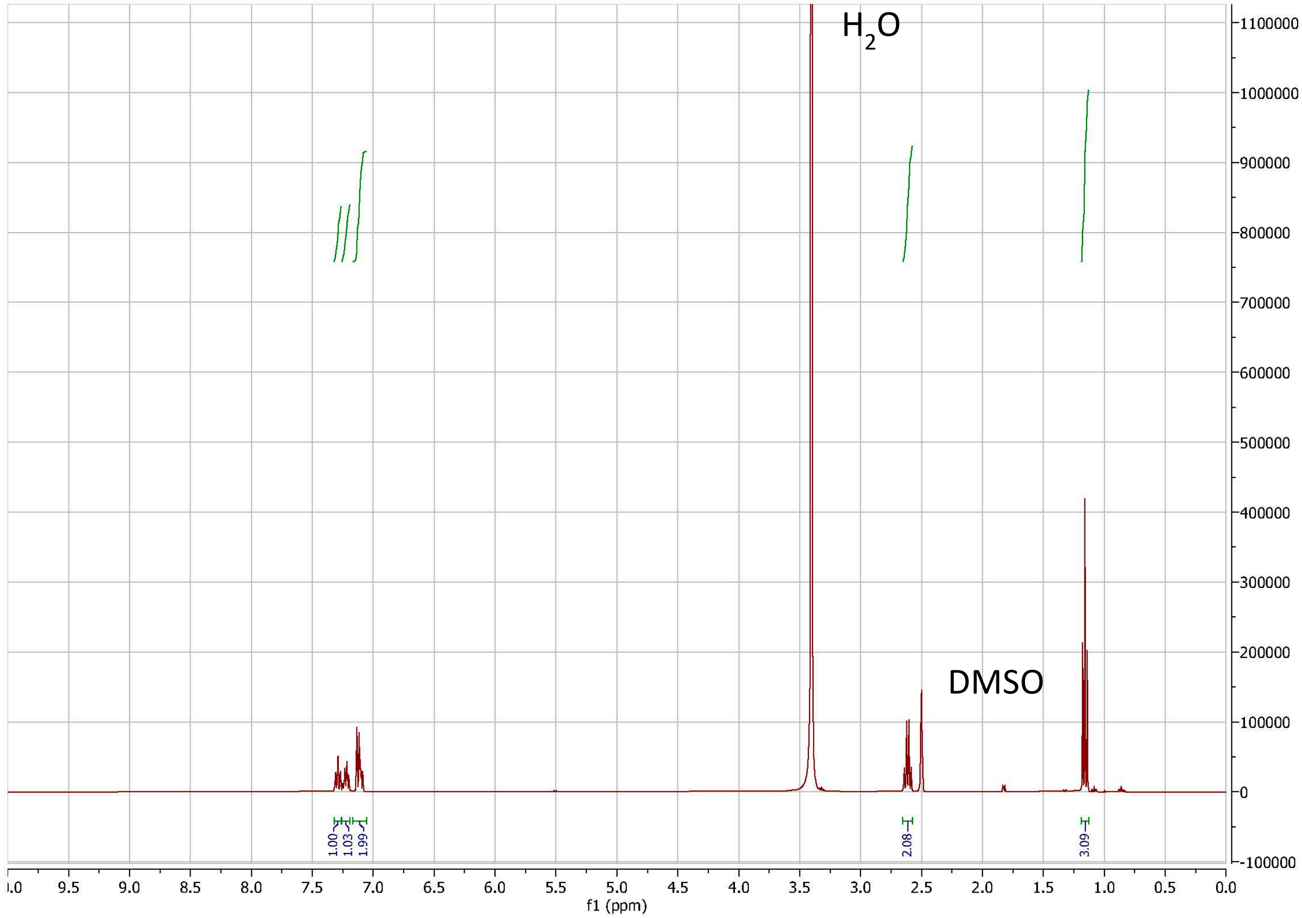

^{1}H NMR 400 MHz $CDCl_3$ δ 1H NMR (400 MHz, DMSO) δ 7.29 (td, J = 7.9, 2.1 Hz, 1H), 7.26 – 7.19 (m, 1H), 7.14 – 7.08 (m, 2H), 2.61 (q, J = 7.6 Hz, 2H), 1.16 (t, J = 7.6 Hz, 3H).

Figure S12. GC/MS of compound **5** (1-ethyl-2-fluorobenzene)

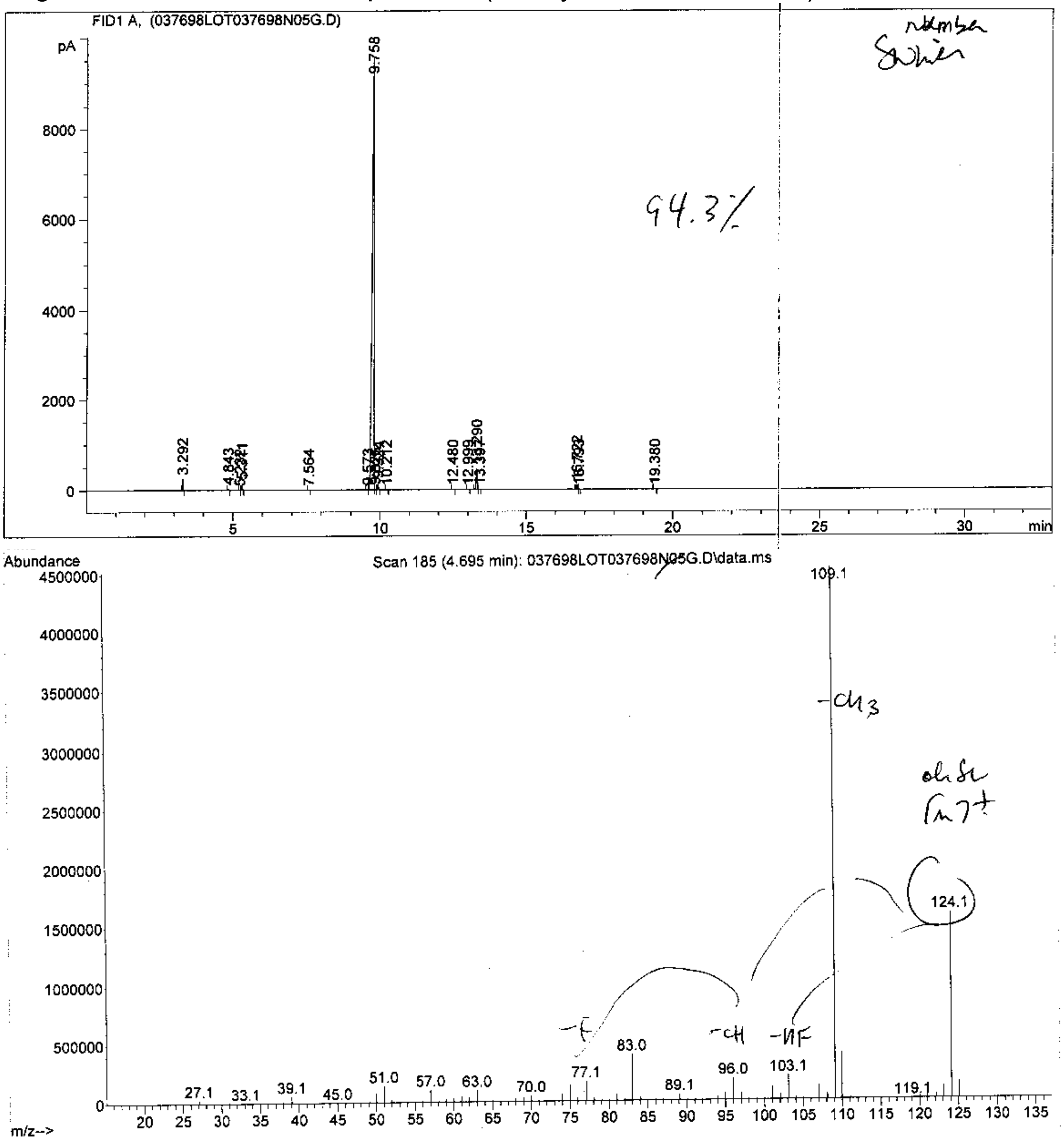

Figure S13. ^{1}H NMR (400 MHz) of compound **7** (1,4-diethyl-2-fluorobenzene)

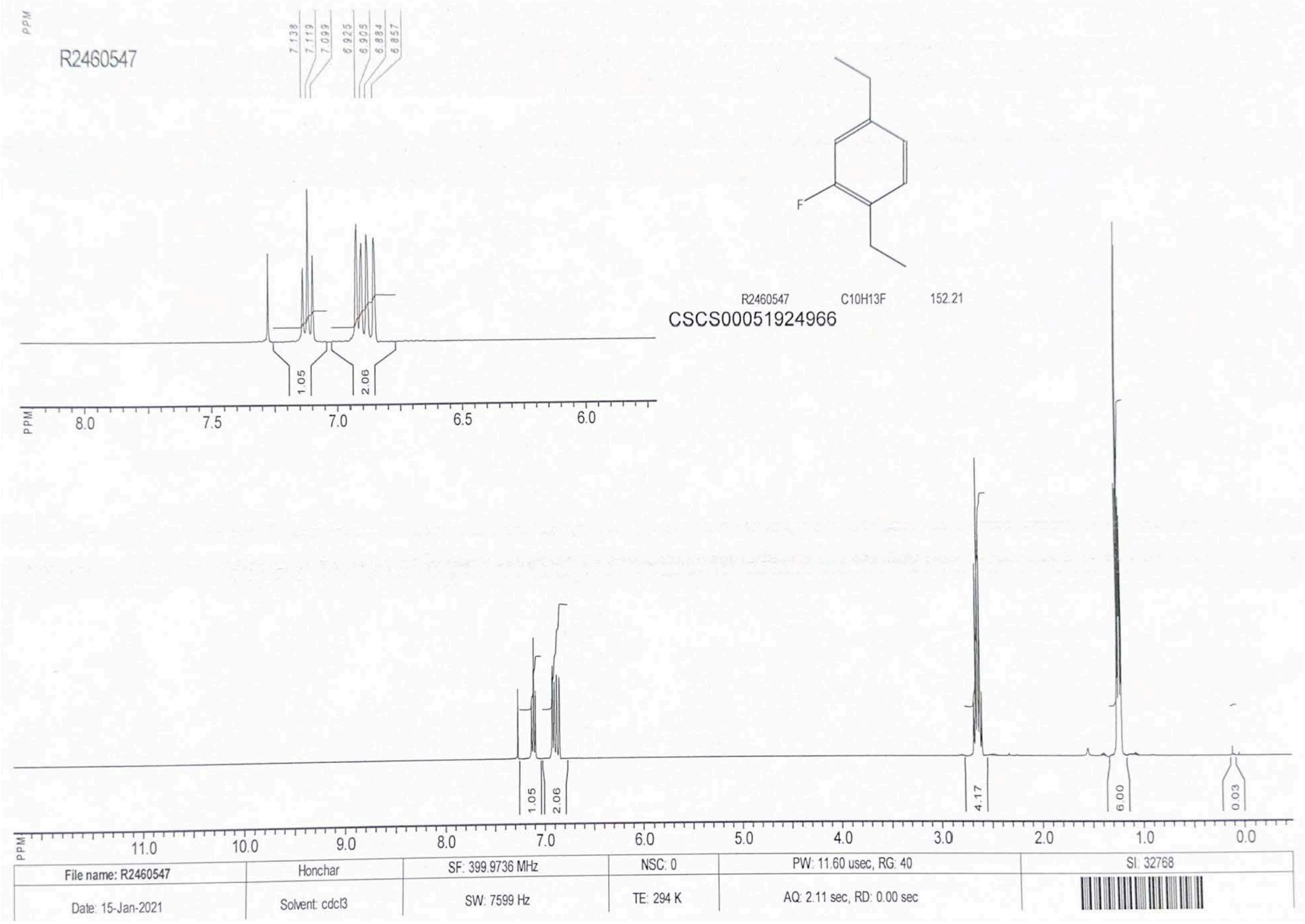

^{1}H NMR 400 MHz $CDCl_3$ δ 1.23 (t, 6H), 2.63 (q, 4H), 6.86-6.88 (d, 1H), 6.6.91-6.93 (d, 1H), 7.10-7.14 (t, 1H).

Figure S14. GC/MS of compound **7** (1,4-diethyl-2-fluorobenzene)

CSCS00051924966

Analysis Report

Agilent | Trusted Answers

Sample Information

Name	R2460547	Data File Path	D:\MassHunter\GCMS\1\data\01_19\R2460547.D
Sample ID		Acq. Time (Local)	20-Jan-21 2:28:35 (UTC+02:00)
Instrument	GCMS-5	Method Path (Acq)	D:\MassHunter\GCMS\1\methods\UNIV_ACN.M
Position	132		

Sample Chromatograms

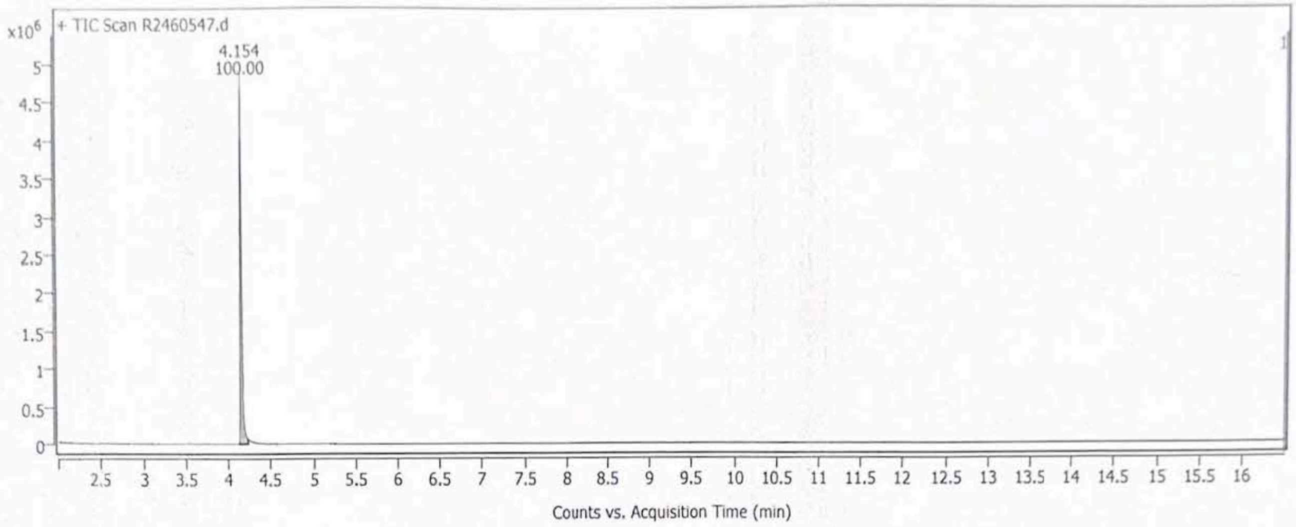

Chromatogram Peaks

Peak	RT	Area	Area Sum %
1	4.154	6946735	100.00

Sample Spectra

+ Scan (rt: 4.14-4.17 min) **Peak 1 from + TIC Scan**

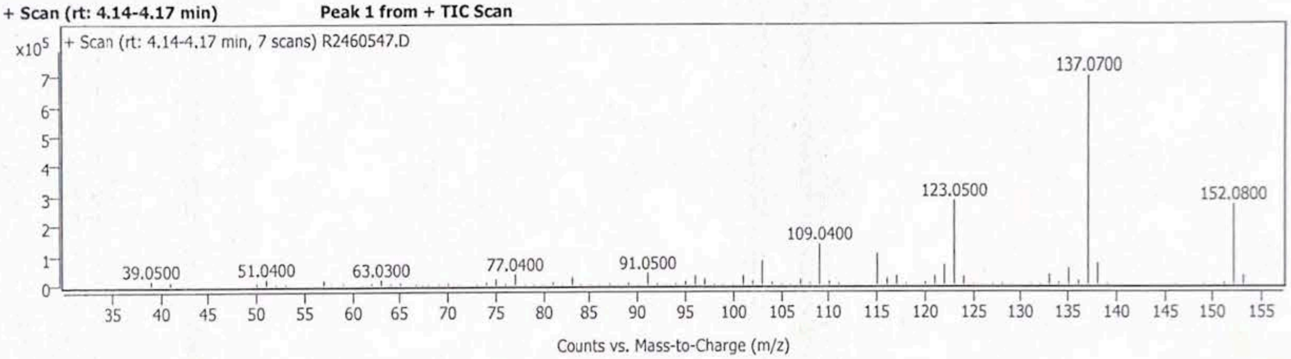

Figure S15. ^{1}H NMR (400 MHz) of compound **8** (1-isopropyl-2-fluorobenzene)

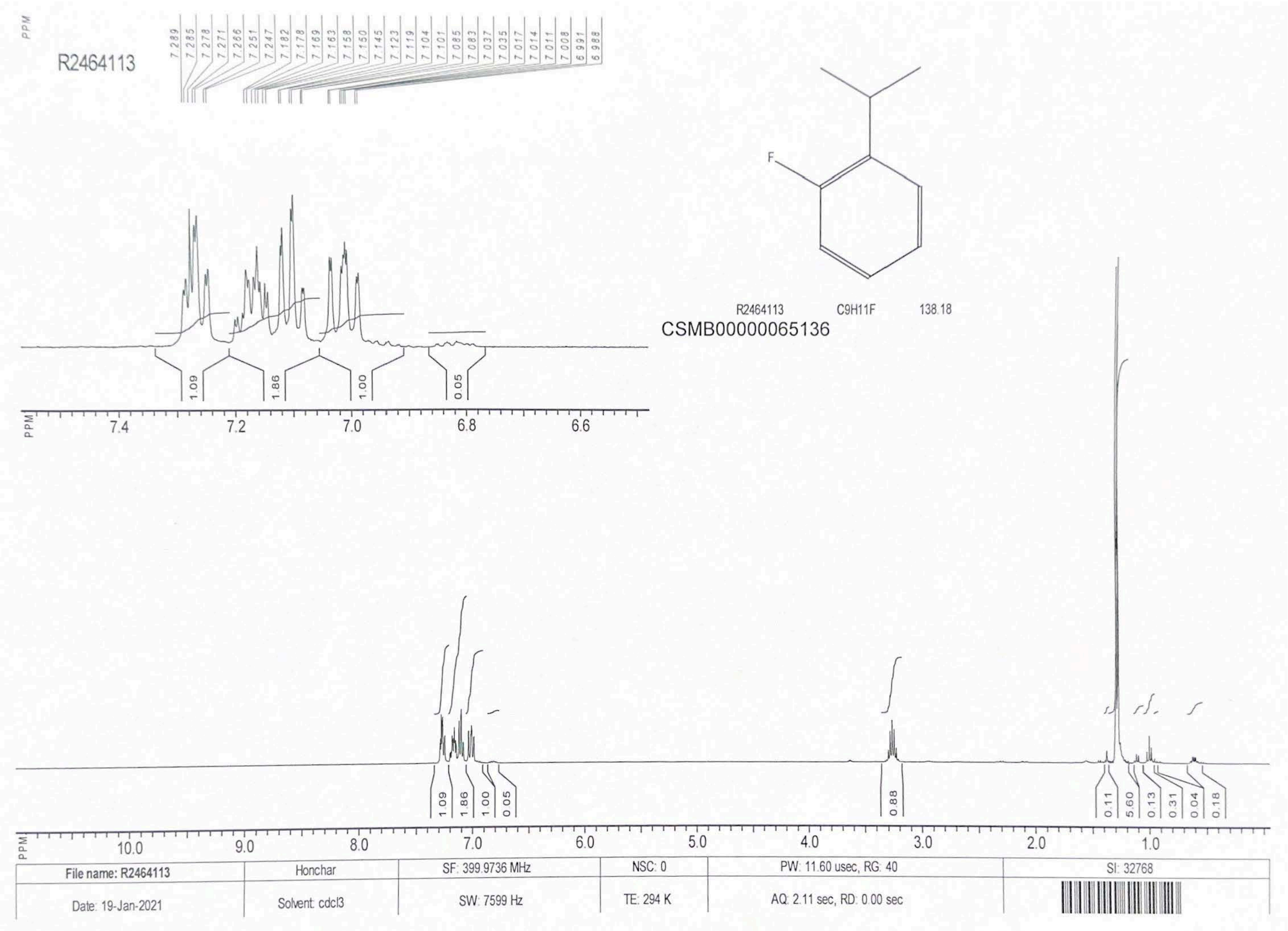

^{1}H NMR 400 MHz $CDCl_3$ δ 1.29 (d, 6H), 3.28 (td, 1H), 6.988-7.035 (td, 1H), 7.073-7.182 (m, 1H), 7.247-7.289 (m, 1H).

Figure S16. GC/MS of compound **8** (1-isopropyl-2-fluorobenzene)

Library Search Report

```
Data File : C:\MSDCHEM\1\DATA\01_21\R2464113.D          Vial: 84
Acq On    : 21 Jan 2021  17:13                       Operator:
Sample    : R2464113                                 Inst    : GCMS2 #1
Misc      : CH3CN                                    Multiplr: 1.00
                                               Sample Amount: 0.00
MS Integration Params: autoint1.e
Method    : C:\MSDCHEM\1\METHODS\UNIVERS.M (Chemstation Integrator)
Title     :
```

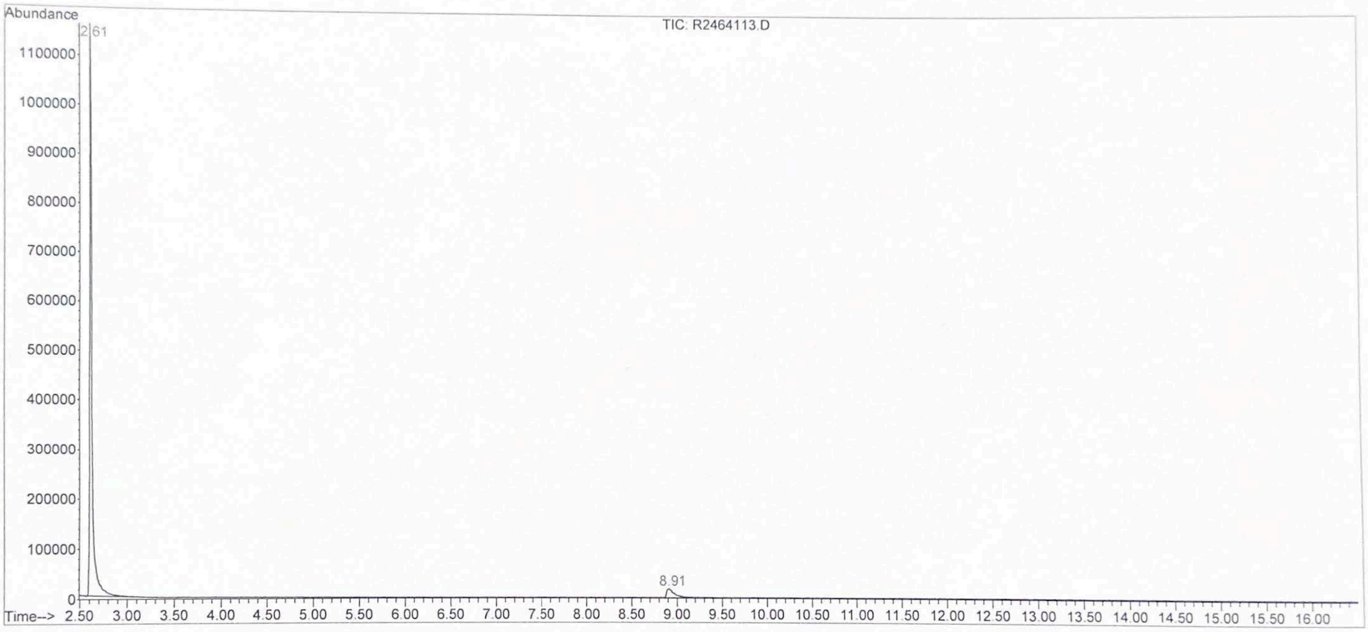

Unknown Spectrum based on Apex

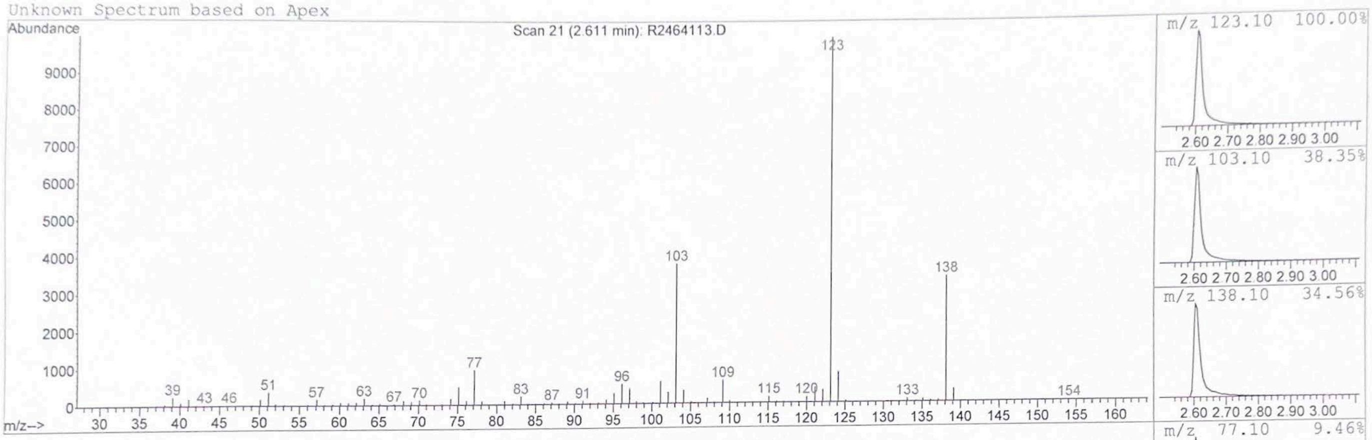

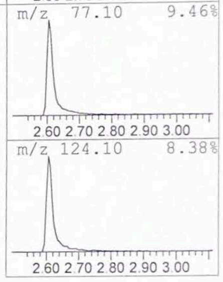


```
Data File: C:\MSDCHEM\1\DATA\01_21\R2464113.D
Sample   : R2464113

Peak Number:  1     at      2.61 min  Area:  18857380  Area % 94.77

CTheDaBabese\EMPTYfrom eaNo librawgre retrieveRef#    CAS#      Qual
--------------------------------------------------------------------
```

Figure S17. ^{1}H NMR (400 MHz) of compound **10** (1,4-diisopropylfluorbenzene)

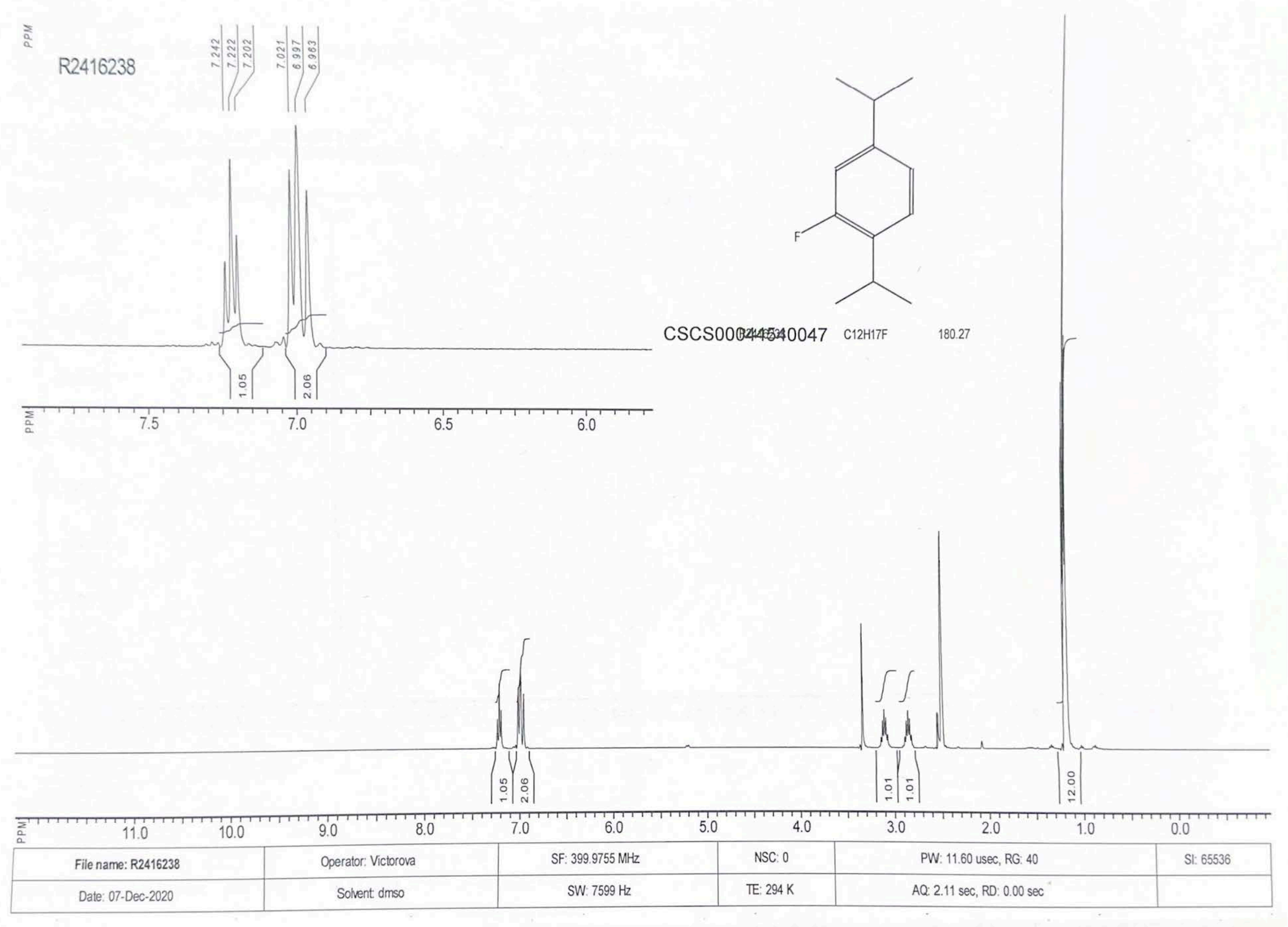

^{1}H NMR 400 MHz DMSO δ 1.19 (t, 12H), 2.87 (sept, 1H), 3.12 (sept, 1H), 6.96-7.02 (t, 2H), 7,20-7.24 9t, 1H).

Figure S18. GC/MS of Compound **10** (1,4-diisopropylfluorbenzene)

Library Search Report

```
Data File : C:\MSDCHEM\1\DATA\12_07\R2416238.D          Vial: 6
Acq On    :  7 Dec 2020  13:27                       Operator:
Sample    : R2416238                                 Inst     : GCMS2 #1
Misc      : CH3OH                                    Multiplr: 1.00
                                               Sample Amount: 0.00
MS Integration Params: autoint1.e
Method    : C:\MSDCHEM\1\METHODS\UNIVERS.M (Chemstation Integrator)
Title     :
```

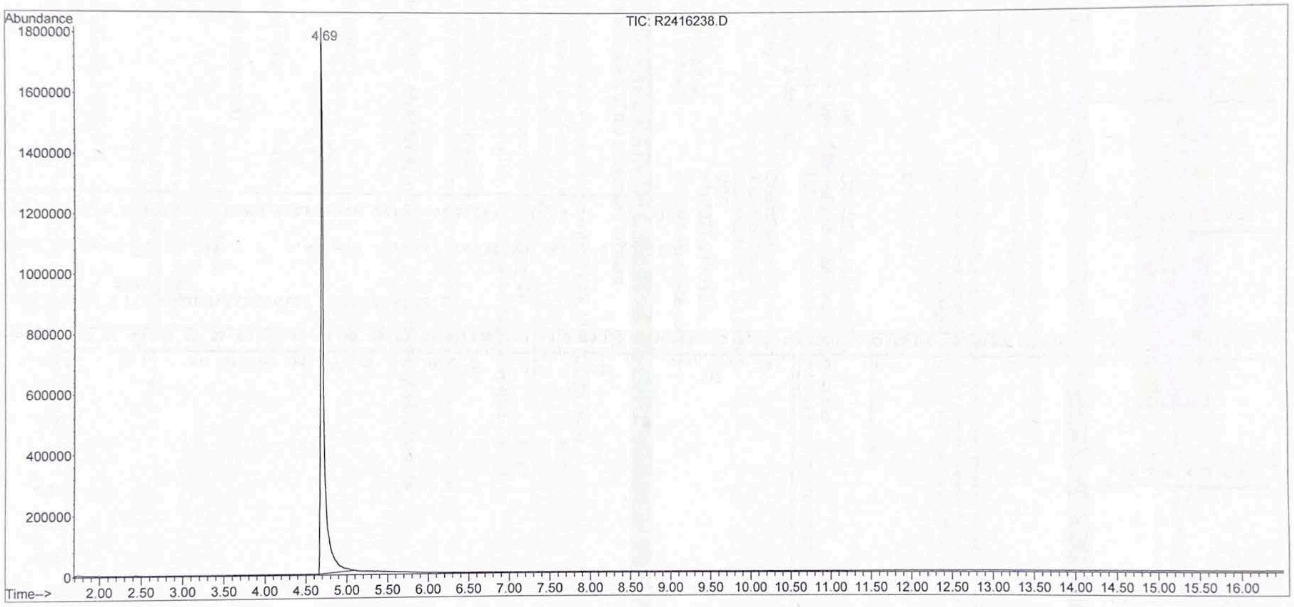

CSCS00044540047

Library Search Report - Chemstation Integrator

Unknown Spectrum based on Apex

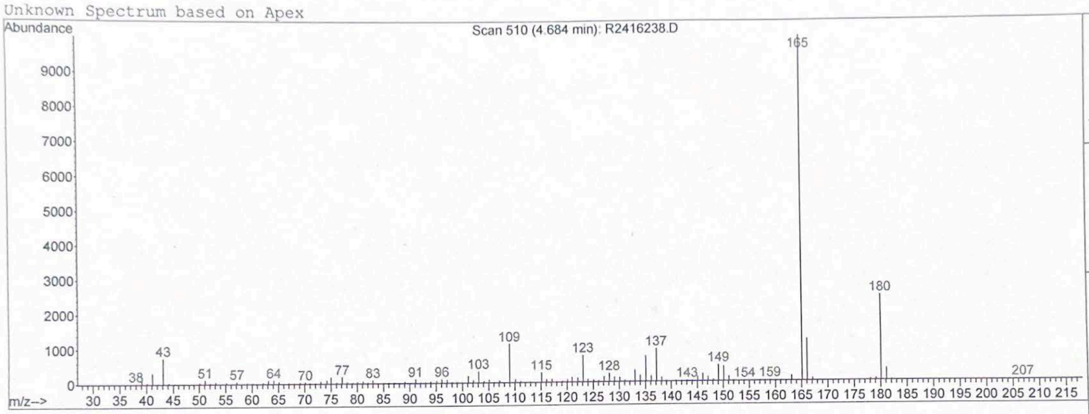

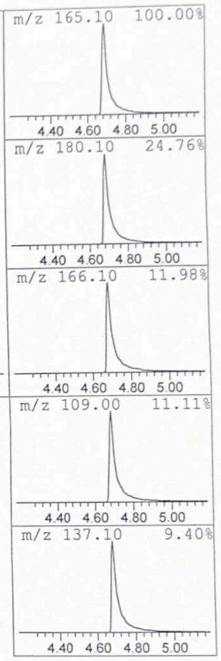


```
Data File: C:\MSDCHEM\1\DATA\12_07\R2416238.D
Sample   : R2416238

Peak Number:  1     at     4.68 min  Area:  44834899  Area % 100.00

CThDaBabase\EMPTYfLom eaNo hibsawgre retrieveRef#    CAS#      Qual
----------------------------------------------------------------------
```

Figure S19. ^{1}H NMR (400 MHz) of compound **11** (1-tertbutyl-2-fluorobenzene)

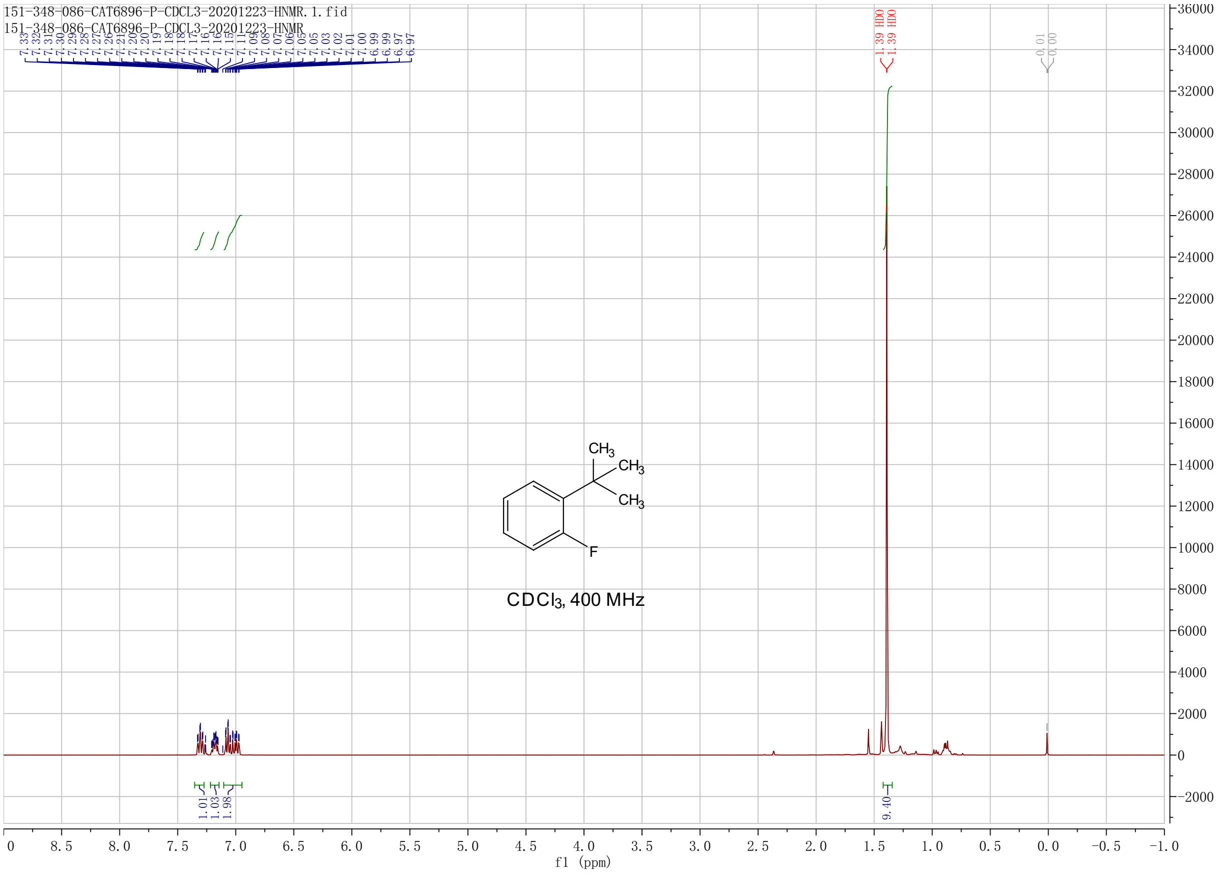

^{1}H NMR 400 MHz $CDCl_3$ δ 1.39 (s, 9H), 1.29 (d, 6H), 3.28 (td, 1H), 6.988-7.035 (td, 1H), 7.073-7.182 (m, 1H), 7.247-7.289 (m, 1H).

Figure S20. HPLC of compound **11** (1-tertbutyl-2-fluorobenzene)

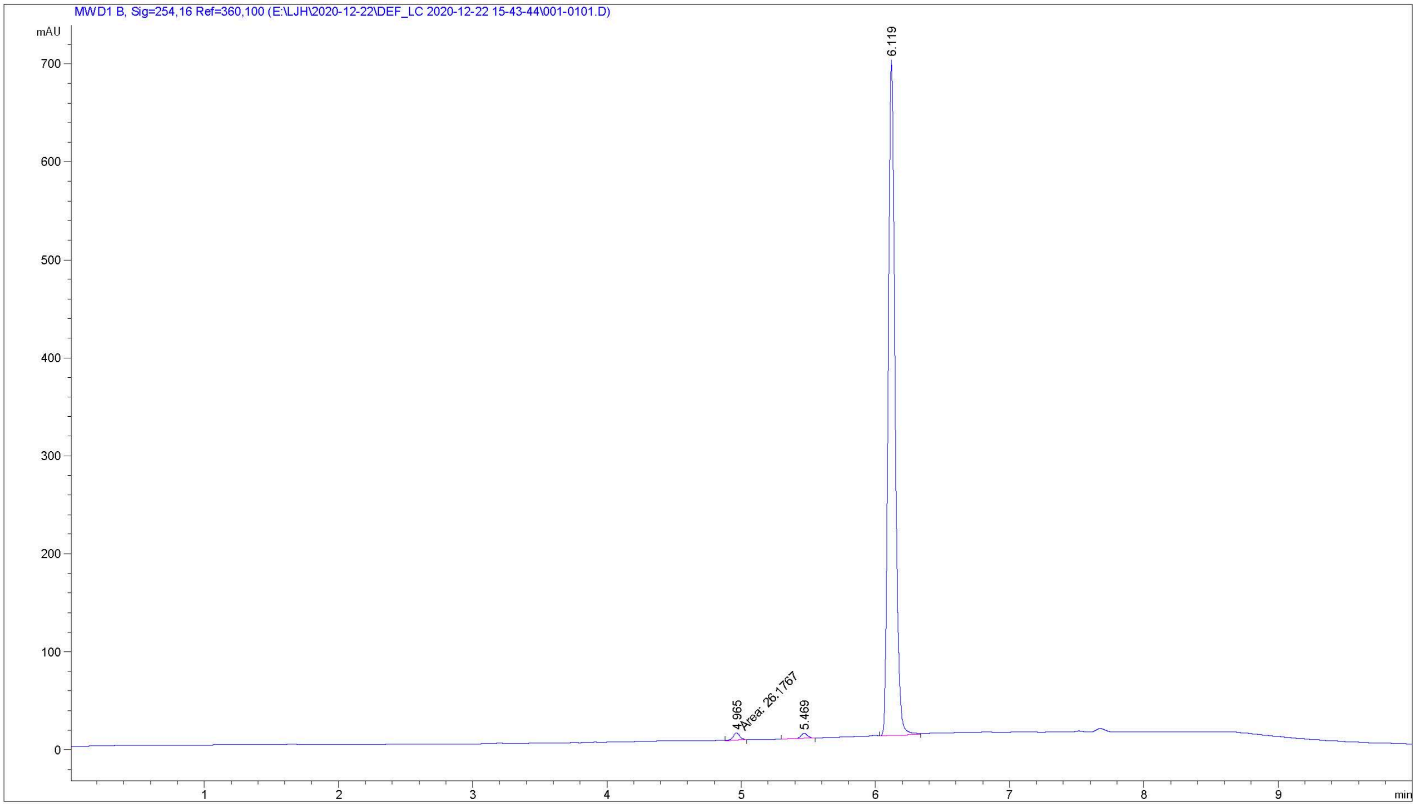

===

Area Percent Report

===

Sorted By : Signal
Multiplier: : 1.0000
Dilution: : 1.0000
Use Multiplier & Dilution Factor with ISTDs

Signal 1: MWD1 B, Sig=254,16 Ref=360,100

Peak #	RetTime [min]	Type	Width [min]	Area [mAU*s]	Height [mAU]	Area %
1	4.965	MM	0.0595	26.17669	7.33804	1.0546
2	5.469	BB	0.0548	17.42636	4.97770	0.7021
3	6.119	BV	0.0553	2438.56128	689.61548	98.2433

Totals : 2482.16433 701.93123

Figure S21. ^{1}H NMR (400 MHz) of compound **13** (1,4-ditertbutyl-2-fluorobenzene)

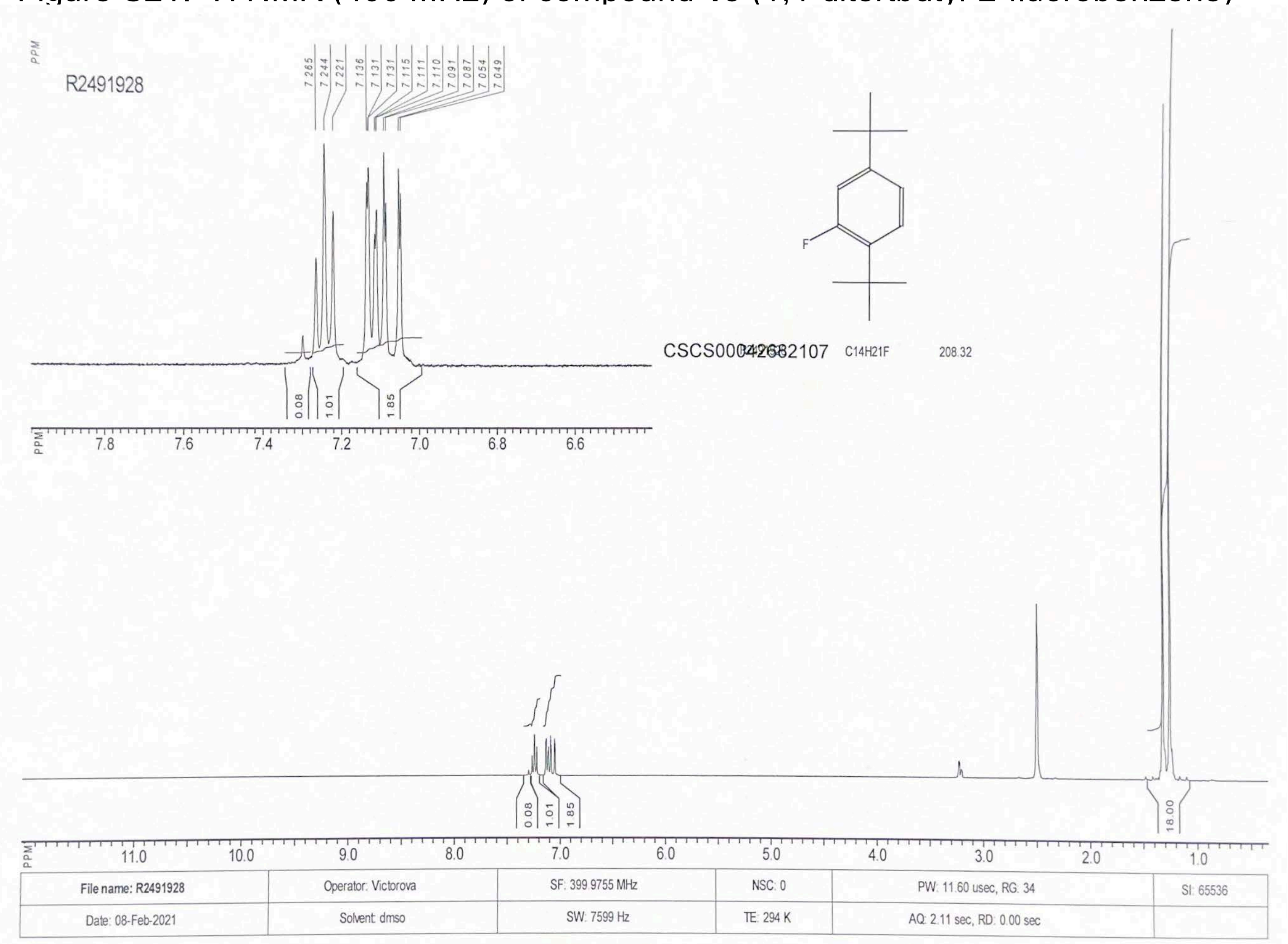

^{1}H NMR 400 MHz DMSO δ 1.25 (s, 9H), 1.41 (s, 9H), 7.07 (dd, 1H), 7.12 (dt, 1H), 7.24 (t, 1H).

Figure S22. GC/MS of compound **13** (1,4-ditertbutyl-2-fluorobenzene)

```
Data File : C:\MSDCHEM\1\DATA\02_10\R2491928.D          Vial: 71
Acq On    : 10 Feb 2021  23:17                      Operator:
Sample    : R2491928                                Inst     : GCMS2 #1
Misc      : CH3CN                                   Multiplr: 1.00
                                             Sample Amount: 0.00

MS Integration Params: autoint1.e
Method    : C:\MSDCHEM\1\METHODS\UNIVERS.M (Chemstation Integrator)
Title     :
```

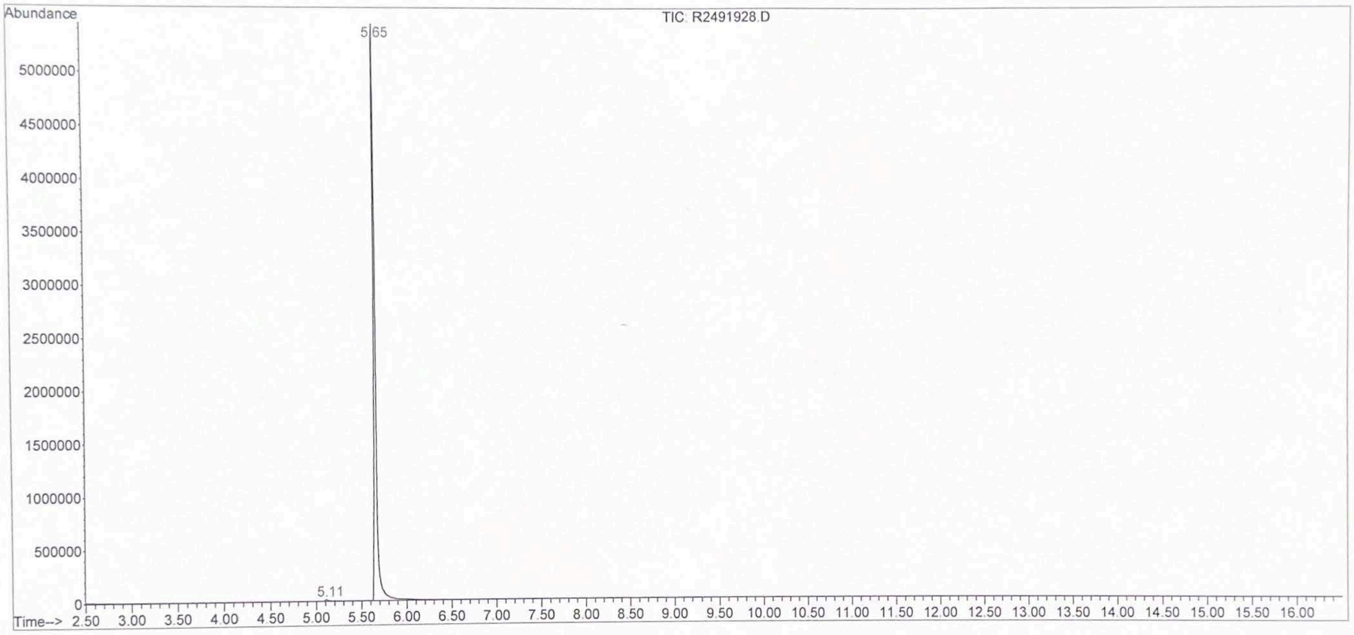

Unknown Spectrum based on Apex

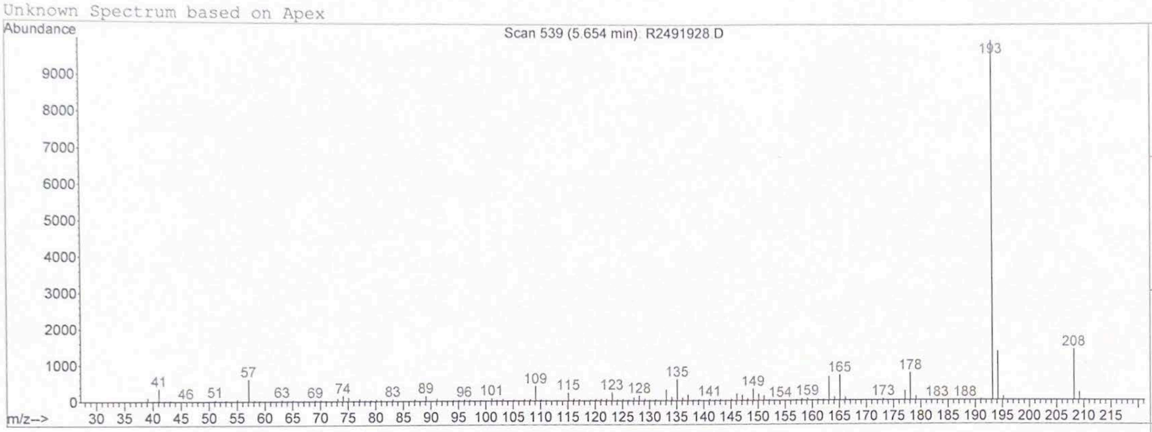

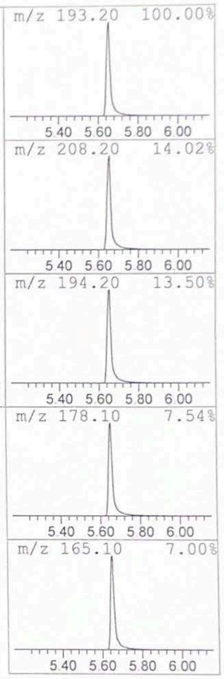


```
Data File: C:\MSDCHEM\1\DATA\02_10\R2491928.D
Sample   : R2491928

Peak Number:  2     at      5.65 min  Area:  85578976  Area % 99.67

CThDaBabase\EMPSYfrom eaNb hibsawgre retrieveRef#    CAS#      Qual
----------------------------------------------------------------------
```

Figure S23. ^{1}H NMR (400MHz) of compound **6** (1,3-diethyl-2-fluorobenzene)

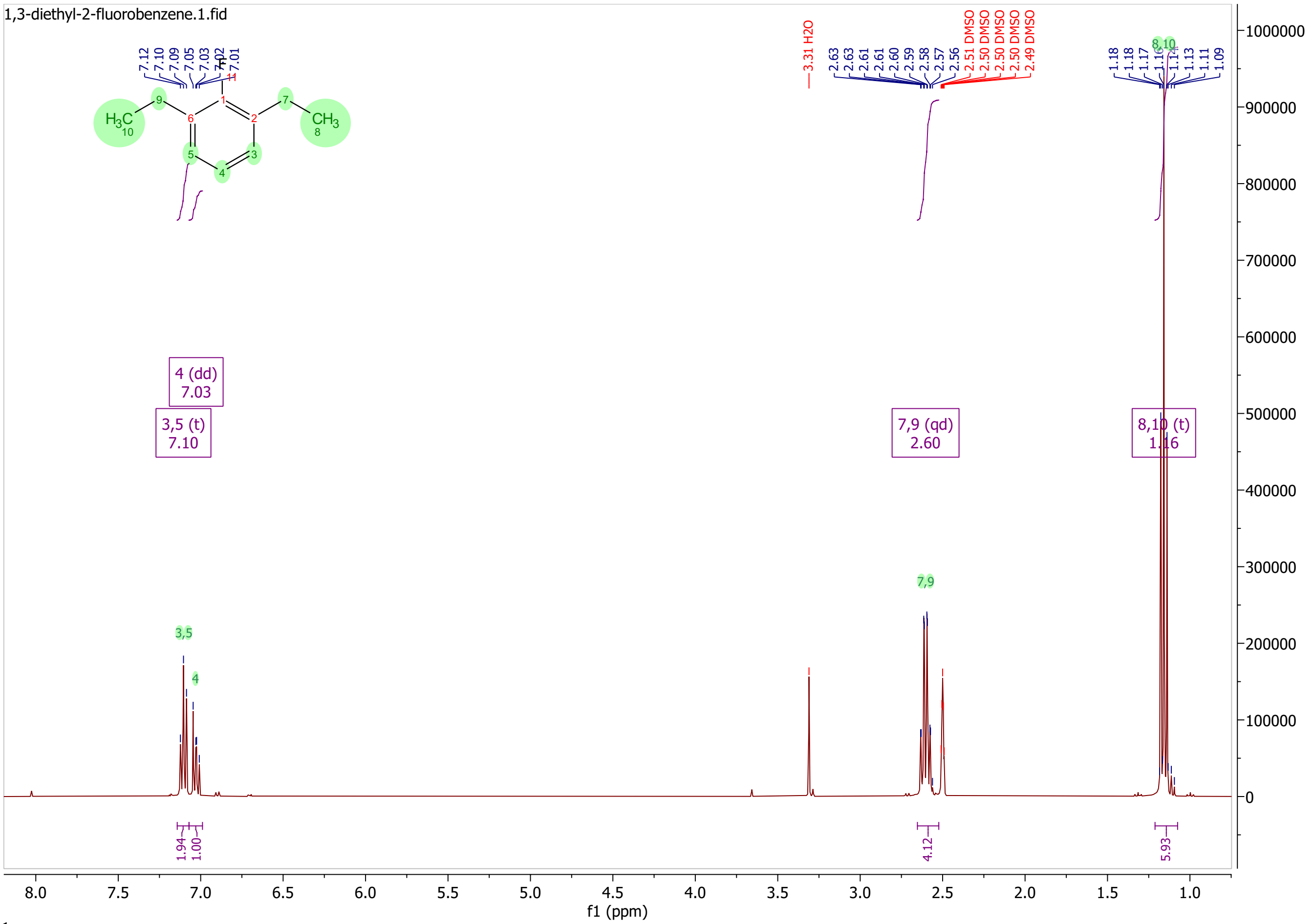

^{1}H NMR 400 MHz DMSO δ 1.16 (t, 6H), 2.56-2.63 (qd 4H), 7.03 (dd, 1H), 7.10 (t, 2H)

Figure S24. HPLC of compound **6** (1,3-diethyl-2-fluorobenzene)

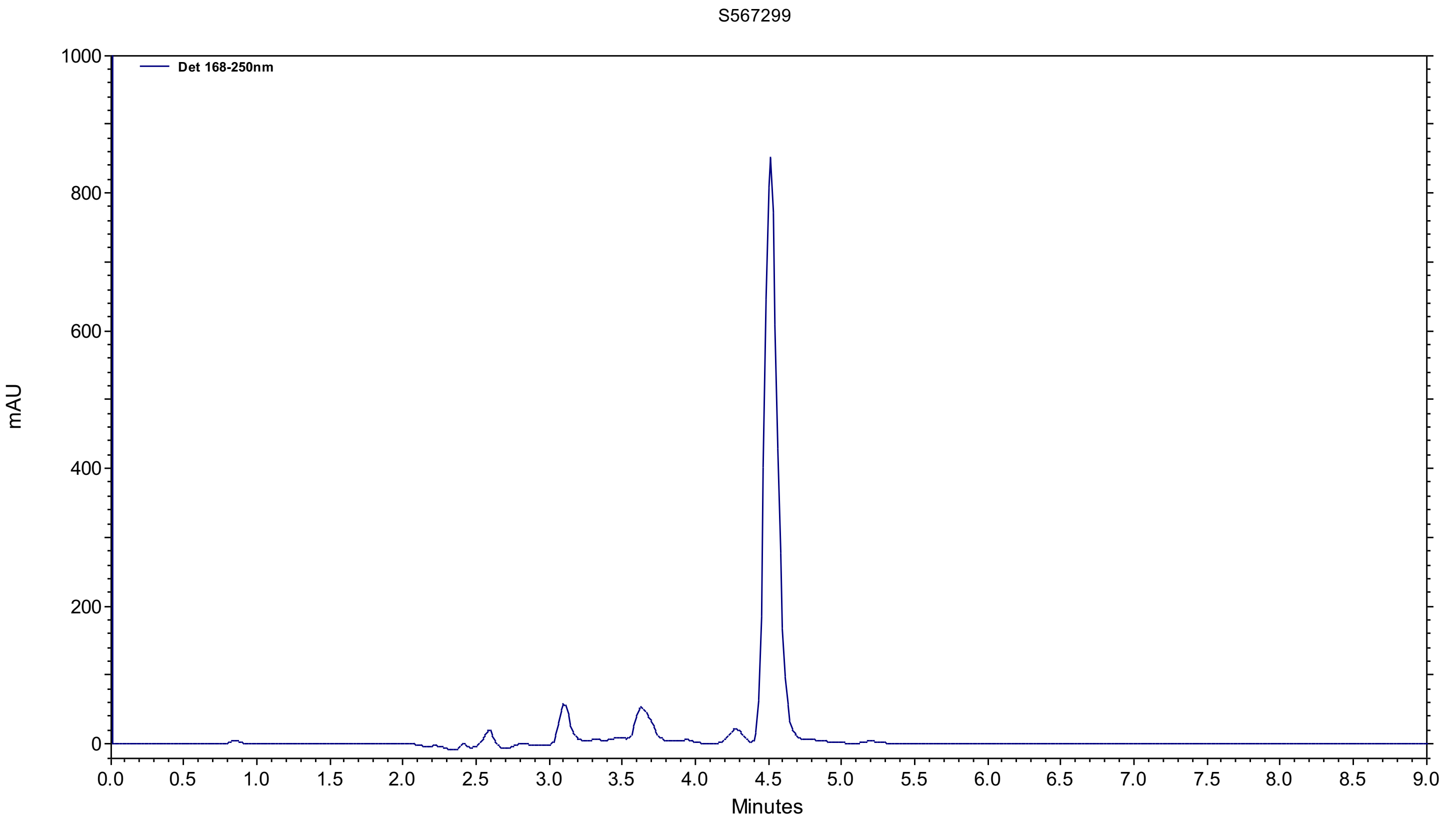

Solvent: HCN:MeOH (volume ratio 80:20), Flow rate: 1ml/min

Inject: 10ul S567299

five peaks @250 nm (2.6min, 3.1 min, 3.6min, 4.3min, 4.5min)

Major peak: 4.5min

Peak area: 36@2.6min, 97@3.1min, 81@3.6min, 27@4.3min, 5975@4.5min

Purity based on Peak area (total two injections): 96.12% and 95.81%

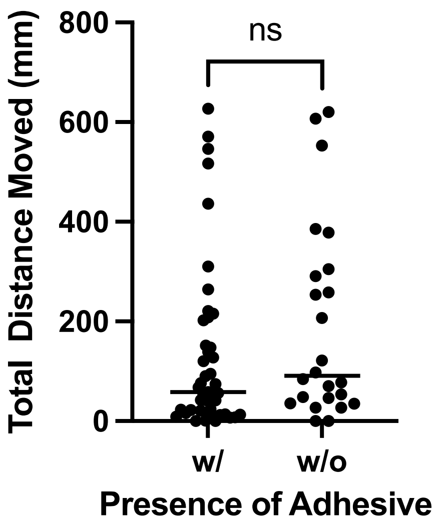

Figure S25. Effect of 3M Adhesive on 5 dpf Zebrafish Movement. In order to create a sealed environment for testing zebrafish behavior with volatile anesthetics, glass 96-well plates were sealed with 3M 300LSE adhesive and inverted prior to observation in the Daniovision chamber. In order to confirm that there were no significant behavioural changes due to the presence of this seal, a comparison was made between this method and a standard open well-plate used in experiments with non-volatile compounds. Spontaneous movement over 10 minutes was recorded for zebrafish in 2% DMSO either in an open glass 96-well plate, or in the same plate sealed with the 3M 300LSE adhesive. No statistical difference was noted between the groups when compared with a Welch's t-test.

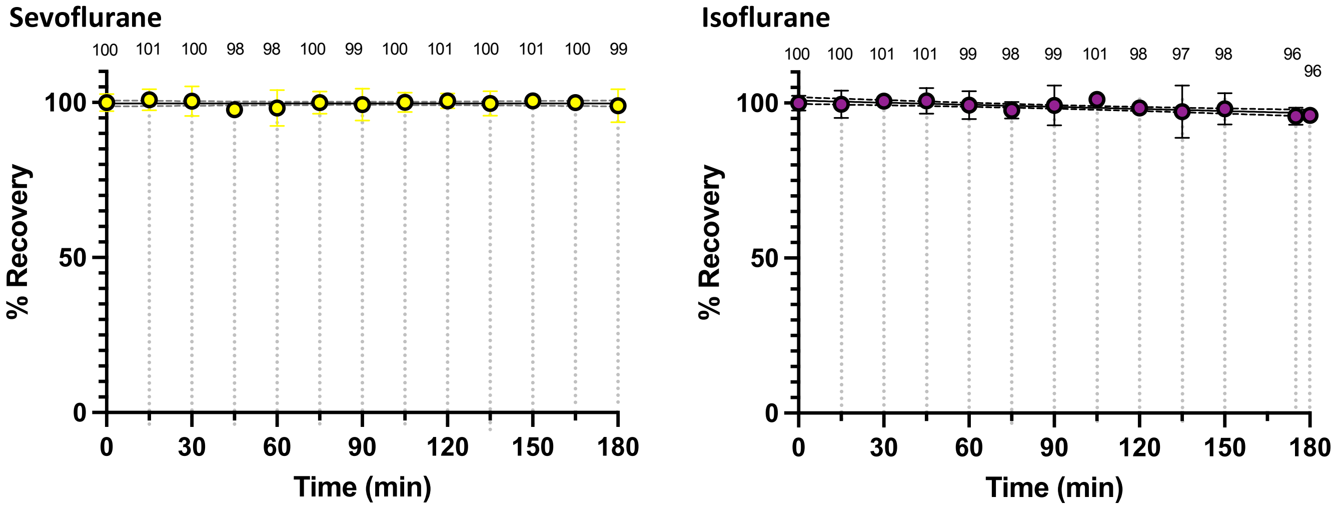

Figure S26. Concentration of Sevoflurane and Isoflurane in the Glass 96-well Plates Over Time. To test the effectiveness of the 3M 300LSE adhesive to prevent loss of the sevoflurane and isoflurane, the concentration was monitored over time by HPLC. The recovery of these anesthetics over 3 hours is shown in the graphs above. Sevoflurane concentration remained constant during the 3-hour testing period, but there was a slow loss of isoflurane over time with 96% of the original concentration remaining at the 3-hour mark. This finding may be due in part to their relative vapor pressures (sevoflurane: 157 mmHg, isoflurane: 238 mmHg at 20°C). Behavioural experiments using this setup occur with a 30-minute incubation period, and thus this method of sealing the plates with an adhesive strip is sufficient for this purpose with both anaesthetics.
